# Luvadaxistat: A Novel Potent and Selective d-Amino Acid Oxidase Inhibitor Improves Cognitive and Social Deficits in Rodent Models for Schizophrenia

**DOI:** 10.1007/s11064-023-03956-2

**Published:** 2023-06-08

**Authors:** Rosa Fradley, Pascal Goetghebeur, David Miller, Russell Burley, Sarah Almond, Agnès Gruart i Massó, José M. Delgado García, Bin Zhu, Eimear Howley, Jo C. Neill, Ben Grayson, Philip Gaskin, Mark Carlton, Ian Gray, Jordi Serrats, Ceri H. Davies

**Affiliations:** 1 Neuroscience Drug Discovery Unit, Takeda, Cambridge, UK; 2 Division of Neurosciences, Pablo de Olavide University, Seville, Spain; 3 Division of Pharmacy and Optometry, School of Health Sciences, University of Manchester, Manchester, UK; 4 Neuroscience Drug Discovery Unit, Takeda California, 9625 Towne Centre Dr, San Diego, CA 92121 USA; 5 Takeda Pharmaceuticals Company Limited, Fujisawa, Kanagawa Japan

**Keywords:** Schizophrenia, Negative symptoms, Cognition, Long-term potentiation

## Abstract

N-methyl-d-aspartate (NMDA) receptor hypofunctionality is a well-studied hypothesis for schizophrenia pathophysiology, and daily dosing of the NMDA receptor co-agonist, d-serine, in clinical trials has shown positive effects in patients. Therefore, inhibition of d-amino acid oxidase (DAAO) has the potential to be a new therapeutic approach for the treatment of schizophrenia. TAK-831 (luvadaxistat), a novel, highly potent inhibitor of DAAO, significantly increases d-serine levels in the rodent brain, plasma, and cerebrospinal fluid. This study shows luvadaxistat to be efficacious in animal tests of cognition and in a translational animal model for cognitive impairment in schizophrenia. This is demonstrated when luvadaxistat is dosed alone and in conjunction with a typical antipsychotic. When dosed chronically, there is a suggestion of change in synaptic plasticity as seen by a leftward shift in the maximum efficacious dose in several studies. This is suggestive of enhanced activation of NMDA receptors in the brain and confirmed by modulation of long-term potentiation after chronic dosing. DAAO is highly expressed in the cerebellum, an area of increasing interest for schizophrenia, and luvadaxistat was shown to be efficacious in a cerebellar-dependent associative learning task. While luvadaxistat ameliorated the deficit seen in sociability in two different negative symptom tests of social interaction, it failed to show an effect in endpoints of negative symptoms in clinical trials. These results suggest that luvadaxistat potentially could be used to improve cognitive impairment in patients with schizophrenia, which is not well addressed with current antipsychotic medications.

**Supplementary Information:**

The online version contains supplementary material available at 10.1007/s11064-023-03956-2.

## Introduction

Schizophrenia is a complex psychiatric disorder marked by positive, negative, and cognitive symptoms. Positive symptoms are most readily recognized and can be controlled by antipsychotic drugs, but there are currently no treatments for negative symptoms or cognitive dysfunction, both of which are persistent and disabling [1]. Different hypotheses for the pathophysiology of schizophrenia have been put forward based on clinical observations and preclinical experiments [2]. One hypothesis suggests that dysregulation of the dopaminergic system underlies the development of positive symptoms, and this has been supported by the efficacy of dopamine receptor antagonists in the clinical treatment of positive symptoms. The glutamatergic hypothesis of schizophrenia has been linked to all three domains [3], and administration of N-methyl-d-aspartate (NMDA) receptor (NMDAR) antagonists induces schizophrenia-like psychosis, social dysfunction, and cognitive impairments in healthy volunteers and exacerbates symptoms in patients with schizophrenia [4–6].

Genome-wide association studies have identified genes relating to the NMDAR signaling complex and glutamatergic neurotransmission to be associated with an increased risk of schizophrenia [7], and molecules that enhance NMDAR signaling, such as d-serine and inhibitors of glycine transporter 1 (GlyT1), have been reported to improve negative and cognitive function in clinical trials [8, 9]. Collectively, these human data suggest that modulation of NMDAR functions is a reasonable clinical approach for the treatment of patients with schizophrenia. NMDARs play a critical role in key aspects of brain function, including modulation of synaptic plasticity, cognitive performance, and synapse formation [10]. Their activation requires the presence of both the agonist glutamate, which binds to the GluN2 and GluN3 subunits, and co-agonists such as glycine or d-serine, which bind to the GluN1 subunit [11, 12]. While hypofunction of the NMDAR is a dominant hypothesis in the pathophysiology of schizophrenia [1, 13, 14], direct agonism of the receptor has not proven to be a successful route to a potential therapy owing to excitotoxicity [15]. Therefore, other approaches to increase functionality of the NMDAR have been explored, such as the use of GlyT1 inhibitors to enhance levels of glycine [16, 17]. This approach has not been successful in the clinic so far as shown by the GlyT1 inhibitor bitopertin. This was evaluated in phase 3 clinical trials for the treatment of negative symptoms of schizophrenia, but showed no evidence of efficacy as an adjunctive treatment [18]. AMG747, another GlyT1 inhibitor, also targeted negative symptoms of schizophrenia and, while significant effects of a single dose on secondary outcomes were found, clinical development of AMG747 did not progress further [17].

An alternative way to increase the functionality of the NMDAR is to enhance levels of D-serine. Although glycine and d-serine are co-agonists of the NMDAR, they have discretely different physiological profiles. d-serine is the more potent co-agonist at the NMDAR [19] and is not present at saturating levels in the brain, unlike endogenous glycine [20]. There is evidence that d-serine acts preferentially on the NR2A subunit of the NMDAR, which is located within the synapse, whereas glycine has high affinity for the NR2B subunit of the NMDAR, which is preferentially found extrasynaptically [14, 21, 22]. As well as being an NMDA-type glutamate receptor co-agonist, d-serine has also been shown to impact synaptic plasticity by acting as an agonist on the cerebellar-restricted glutamate receptor delta2 [23]. Support for a direct role for d-serine in the pathophysiology of schizophrenia is provided by reports of decreased d-serine levels in the cerebrospinal fluid (CSF) and blood of patients with schizophrenia [24–30]. d-serine dosed alone at 60 mg/kg/day has been shown to improve a variety of clinical symptoms in patients, including negative and neurocognitive symptoms of schizophrenia and mismatch negativity [24, 31, 32]. It has also been shown to improve negative, positive, and cognitive symptoms when added to antipsychotics at 30 mg/kg in patients with schizophrenia [33]. However, high doses of d-serine have been shown to be nephrotoxic, both in rats [34] and in patients undergoing clinical trials [31]. Therefore, an alternative to increasing central d-serine levels without the application of high concentrations of exogenous d-serine is required. In the body, d-serine is formed from l-serine via the enzyme serine racemase and subsequently catabolized by the enzyme d-amino acid oxidase (DAAO); therefore, the use of small-molecule inhibitors of DAAO may be a strategy to increase endogenous d-serine levels [35]. In fact, a DAAO knockout mouse has been shown to have increased extracellular d-serine levels and glycine site occupancy, to have enhanced NMDAR function, and to exhibit behaviors suggestive of antipsychotic behavior such as protection against phencyclidine (PCP)-induced effects [36].

We recently discovered the novel, highly selective, potent DAAO inhibitor TAK-831 (luvadaxistat) (4-hydroxy-6-[21]pyridazine-3(2H)-one) that has the desired attributes for a central nervous system drug. The studies described here investigated whether administration of luvadaxistat elevates d-serine levels in the central nervous system, especially in the cerebellum where DAAO is highly expressed [35]. We also investigated its ability to ameliorate deficits in animal models for cognitive and negative symptoms of schizophrenia and to modulate synaptic plasticity measures such as long-term potentiation.

## Materials and Methods

### Compounds

Luvadaxistat [37] was synthesized by Takeda Cambridge Ltd (Cambridge, UK) and suspended in 1% TWEEN 80/0.5% methylcellulose and administered orally (p.o.), at the doses stated in the experimental designs, at a dose volume of 10 mL/kg for mice and 1 mL/kg for rats. Luvadaxistat was always dosed to animals 5 h before testing, unless otherwise stated. Thioperamide was synthesized by Takeda Cambridge Ltd and dosed in 10% dimethyl sulfoxide/10% polyethylene glycol/10% cremophor/70% distilled water (dH_2_O) at 10 mg/kg intraperitoneally (i.p.)*,* in a dosing volume of 10 mL/kg.

PCP was purchased from Sigma-Aldrich (St Louis, MO, USA) and dosed in dH_2_O at 2 mg/kg i.p*.* twice daily for 7 days in a dosing volume of 1 mL/kg. Risperidone was purchased from Sigma-Aldrich, UK and dosed in pH-buffered dH_2_O at 0.1 mg/kg i.p. in a dosing volume of 1 mL/kg. Haloperidol was purchased from Sigma-Aldrich, UK and dosed in pH-buffered dH_2_O at 0.2 mg/kg p.o. for 21 days (1 h pre-test) in a dosing volume of 1 mL/kg. Scopolamine was purchased from Sigma-Aldrich, UK and dosed in dH_2_O at 0.3 mg/kg subcutaneously in a dosing volume of 10 mL/kg. Polyinosinic-polycytidylic acid (poly[I:C]) was purchased from Sigma-Aldrich, UK and dosed in buffered saline (0.9% sodium chloride) at 5 mg/kg intravenously (i.v.) in a dosing volume of 5 mL/kg.
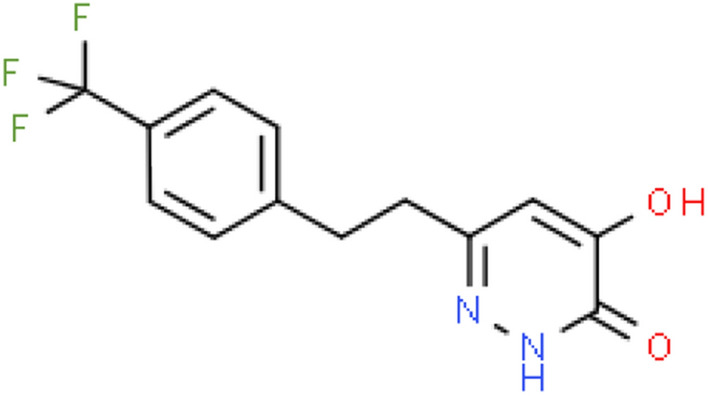


Luvadaxistat (http://www.chemspider.com/Chemical-Structure.29419992.html; accessed Apr 27, 2023).

### Animals

Specific details of animals used are described in the text of each test. Choice of strain, age, and gender for each test was on a case-by-case basis, using previous knowledge of suitable validation data. Animals were housed with food and water available ad libitum. All procedures were performed in accordance with local guidelines (UK and India: Animals [Scientific Procedures] Act 1986; Singapore: Institutional Animal Care and Use Committee protocols; Spain: Spanish BOE 34/11370-421, 2013 and European Council Directive 2010/63/EU for the use of laboratory animals in chronic experiments). All dose groups were pseudo-randomized as part of the experimental design.

### Inhibitory Potency of Luvadaxistat

The methodology has been described previously [38]. Briefly, functional activity of luvadaxistat on the DAAO enzyme was determined by measuring the production of hydrogen peroxide (H_2_O_2_) using the Amplex® Red (Invitrogen, Waltham, MA, USA) detection method as described by Howley, Bestwick [38] and Brandish, Chiu [39]. DAAO activity was detected upon addition of d-serine at a concentration equivalent to its Michaelis constant (1.2 mM, in-house data), and suppression of this response was observed with the application of an inhibitor: in this case, luvadaxistat.

### Enzyme Occupancy

Male C57BL/6J mice weighing 20–30 g were used. Studies were run either at Takeda Cambridge Ltd (animals purchased from Charles River Laboratories, Wilmington, MA, USA) or at Suven Life Sciences Ltd (Hyderabad, India). PGM019260 [38] was used as a tracer compound to measure enzyme occupancy of DAAO. PGM019260 was dissolved in 10% dimethyl sulfoxide/90% hydroxypropyl-beta-cyclodextrin and dosed at 60 μg/kg i.v., in a dose volume of 5 mL/kg via the tail vein 2 h after dosing of luvadaxistat. Mice were sacrificed by decapitation 20 min after tracer administration. Trunk blood was collected for compound analysis, the brain was removed, and the cerebral cortex and cerebellum dissected on ice. Plasma and tissue samples were frozen on dry ice and stored at − 80 °C. Compound levels were quantified by liquid chromatography with tandem mass spectrometry (LC–MS/MS).

#### Enzyme Occupancy Calculation

DAAO enzyme occupancy was measured using the ratio method, where the ratio of total binding to the nonspecific binding of the tracer was generated. The cerebellum represented an area of high enzyme density and, hence, total binding, and the cortex an area of low enzyme density and, hence, nonspecific binding. We used the following equation:

$$\% \mathrm{occupancy} =100 \times \left(1 -\left[\left({\mathrm{ratio}}_{t} - 1\right)/\left({\mathrm{ratio}}_{c} -1\right)\right]\right)$$

where ratio_*t*_ is the ratio of tracer concentration in the cerebellum to that in the cortex in animals pre-treated with test compound, and ratio_c_ is the mean ratio of tracer concentration in the cerebellum to that in the forebrain in vehicle-treated animals.

### d-Serine Measurements

#### Time Course of d-Serine in Cerebellum

Male Wistar rats weighing 240–280 g were used. Studies were run at Takeda Singapore Pte Ltd (animals purchased from the Biological Resource Centre [BRC], Agency for Science, Technology and Research [A*STAR], Singapore). Rats were administered compound and were sacrificed by decapitation at 2, 6, 10, or 24 h post-dose. Cerebella were dissected, and tissue samples were frozen on dry ice and stored at − 80 °C until analysis using LC–MS/MS.

#### Measurements of d-Serine in Plasma, CSF, and Cerebellum

Male Wistar rats approximately 8 weeks of age were used. Studies were run at Takeda Cambridge Ltd (animals purchased from Charles River Laboratories). Rats were administered compound and were sacrificed at 6 h post-dose. Blood, tissue, and CSF were removed, frozen on dry ice, and stored at − 80 °C until analysis using LC–MS/MS. Samples were sent to Sumika Chemical Analysis Service Ltd (Osaka, Japan) to measure d-serine levels using LC–MS/MS.

### Novel Object Recognition Test

Male C57BL/6J mice (aged 7–8 weeks) were used. Studies were run at Takeda Cambridge Ltd (animals purchased from Charles River Laboratories). Mice were dosed acutely or chronically (for 14 days) with luvadaxistat and assessed for their ability to differentiate between novel and previously seen objects after a 3 h inter-trial interval. The day before testing, all animals were allowed to habituate to the testing box for 30 min. On the day of the experiment, mice were submitted to two trials with an inter-trial interval of 3 h. During the acquisition trial (T1), mice were placed in the arena with two identical objects and allowed to explore freely for 5 min. For the retention trial (T2), one of the objects was replaced by an unknown object (novel object), the mice placed back in the arena for 5 min, and the exploration time recorded. For both T1 and T2, the active exploration time was recorded. A discrimination index (D2) was then calculated:

$$\frac{D2 =(\mathrm{time}\,\, {\rm exploring}\,\, {\rm novel }\,\left[T2\right]-\mathrm{time} \,\,{\rm exploring}\,\, {\rm familiar} \,\left[T1\right]}{\mathrm{Overall}\,\, {\rm time}\,\, {\rm exploring}\,\, {\rm objects}\, [T1 +T2]}$$

Data from this study are presented both as a statistical difference between compound-treated animals and vehicle-treated animals and as the D2 score to zero, where zero shows no preference between the novel and familiar objects.

### Attentional Set-Shifting Task Assay

Female Lister hooded rats (aged 12–24 weeks) weighing 230–295 g at the time of testing were used. Studies were run at the University of Manchester, UK (animals purchased from Charles River Laboratories, Wilmington, MA, USA). Both choice of animal model and test set-up are described elsewhere [40, 41]. In brief, animals were dosed sub-chronically (for 7 days) with PCP and then allowed a 7-day washout period to induce a cognitive deficit (see [42] for full overview). Rats were then dosed acutely (study 1) or chronically (for 14 days) (study 2) with luvadaxistat and assessed for their set-shifting ability. In study 3, animals were dosed concurrently for 21 days with haloperidol (0.2 mg/kg) and for 14 days with luvadaxistat, with luvadaxistat treatment starting 7 days after the start of haloperidol dosing.

Data from these studies are presented as mean trials to criterion (definition of ‘criterion’ as described elsewhere [40, 41]) and analyzed by a repeated measures two-way analysis of variance (ANOVA) using phase as a within-subjects factor and drug treatment as a between-subjects factor. Where a significant effect was detected, a one-way ANOVA was then performed followed by a Dunnett’s post hoc *t*-test in order to compare treatment groups versus the appropriate control.

### Classical Eyeblink Conditioning of Behaving Mice Using a Delay Paradigm

Male C57Bl6 mice (aged 12 weeks) were used in the delay eyeblink conditioning (dEBC) study. Studies were run at the animal house facilities of the Pablo de Olavide University, Seville, Spain (animals purchased from Charles River Laboratories). Animals were habituated to the apparatus for 2 days before undergoing 10 days of conditioning. Conditioning involved the pairing of a conditioning stimulus (CS—a tone) with an unconditioned stimulus (US—periorbital electrical stimulus), leading to a blinking reflex. In the dEBC paradigm, the CS and US overlap and co-terminate. Classical conditioning of this reflex to the CS predicts the US and is a basic form of associative learning. Animals then underwent 5 days of extinction, during which the CS was no longer paired with the US, and the conditioned response was lost over time. Animals were assessed for changes in the rate of acquisition after chronic administration (10 days) of luvadaxistat 0.1 and 1 mg/kg during the conditioning period. In a second study, a deficit in the rate of acquisition was induced through the use of scopolamine 0.3 mg/kg during the conditioning period and luvadaxistat 0.1 and 1 mg/kg was administered chronically to determine any reversal. Surgical preparation and recording, and stimulation procedures were carried out as described elsewhere [43].

### Social Interaction in BALB/c Mice

Male BALB/c mice (aged 9–10 weeks) were used. The BALB/c mouse strain has high susceptibility to stress and is thus an effective model for depression [44]. Studies were run at Takeda Singapore Pte Ltd (animals purchased from BRC, A*STAR). Mice were dosed acutely or chronically (for 14 days) with luvadaxistat and assessed for their levels of social interaction (SI). Mice were placed in an arena to habituate for 2 min with two identical cylinders, one at either end of the arena. A normal, age-matched C57BL/6 ‘intruder’ was then placed in one cylinder, and time spent investigating (sniffing) the two cylinders was scored for 5 min. The social preference was computed as follows:

$$\frac{\mathrm{Social} \,\,{\rm preference}=(\mathrm{intruder}\,\, {\rm investigation }-\mathrm{empty} \,\,{\rm investigation})}{\mathrm{Intruder }\,\, {\rm investigation }+\mathrm{empty}\,\, {\rm investigation}}$$

### Social Interaction in Poly(I:C)-Treated Mice

Studies were run at Takeda Cambridge Ltd using female C57BL/6 pregnant dams (Charles River Laboratories) that were treated with poly(I:C) 5 mg/kg i.v. on gestational day 17, causing maternal immune activation and resulting in offspring that show deficits in SI. Male mice from the poly(I:C)-treated dams at 15–16 weeks of age were dosed acutely with luvadaxistat and assessed for their levels of SI using the same protocol as that used for the BALB/c mice. The social preference was computed as above.

### Long-Term Potentiation (LTP) Measurement After In Vivo Dosing in Wild Type Mice

Male mice C57Bl/6J (5–6 weeks old) provided by Charles River Laboratories (Saint Germain sur l’Arbresle, France) (*n* = 97) were orally dosed acutely or sub-chronically (for 14 days) with luvadaxistat at doses of 0.1, 1, and 10 mg/kg, or 0.001, 0.01, 0.1, and 10 mg/kg, respectively. Hippocampal slices were harvested 5 h after dosing; cerebellum and blood plasma were also obtained to measure d-serine levels in the experimental animals. Electrophysiological recordings were obtained from a single hippocampal slice placed on the chamber and perfused with artificial CSF (aCSF) at a constant rate. Extracellular field excitatory postsynaptic potentials (fEPSP) were recorded in the CA1 stratum radiatum using a glass micropipette filled with aCSF. fEPSPs were evoked by electrical stimulation of the Schaffer collaterals/commissural pathway at 0.1 Hz with a glass stimulating electrode placed in the stratum radiatum. To test the effect of luvadaxistat on basal synaptic transmission, input/output curves were constructed at the beginning of the experiment. The slope of fEPSPs was measured and plotted against different intensities of stimulation (from 0 to 100 uA). LTP was induced by a theta burst stimulation protocol (10 bursts of 4 pulses at 100 Hz, with 200 ms between bursts) at baseline stimulation intensity. Following this conditioning stimulus, a 1 h test period was recorded where responses were elicited by a single stimulation every 10 s (0.1 Hz) at the same stimulus intensity.

### Statistics

One-way ANOVAs, two-way ANOVAs, and appropriate post hoc tests were applied when appropriate, and their use is referenced in the results section. Data are expressed as mean ± standard error of the mean. Where this is not the case, it is described in the results section. Statistical significance was set as *P* < 0.05. For behavioral studies, n represents the number of mice per group and sample sizes were determined on the basis of previous studies using similar experimental protocols.

## Results

### In Vitro/Ex Vivo Properties of Luvadaxistat

In vitro, luvadaxistat inhibited oxidative deamination of d-serine via the human recombinant DAAO enzyme in a concentration-dependent manner with a 50% inhibitory concentration (IC_50_) of 14 nmol/L, showing high potency at the target. In a cellular assay using Chinese hamster ovary cells expressing recombinant human DAAO, luvadaxistat inhibited the formation of H_2_O_2_, the co-product of d-serine catalysis, with an IC_50_ of 12 nmol/L; a value almost identical to that measured with the isolated enzyme preparation. In Chinese hamster ovary cells expressing recombinant mouse DAAO, luvadaxistat inhibited H_2_O_2_ formation with an IC_50_ of 5.1 nmol/L, a value in good agreement with its activity on the human DAAO enzyme and further supporting its testing in vivo. Luvadaxistat showed very little off-target activity when profiled in a comprehensive selectivity panel of enzyme and radioactivity binding (data not shown); as such, all physiological effects are considered to be through mechanism of action on DAAO.

**Fig. 1:**
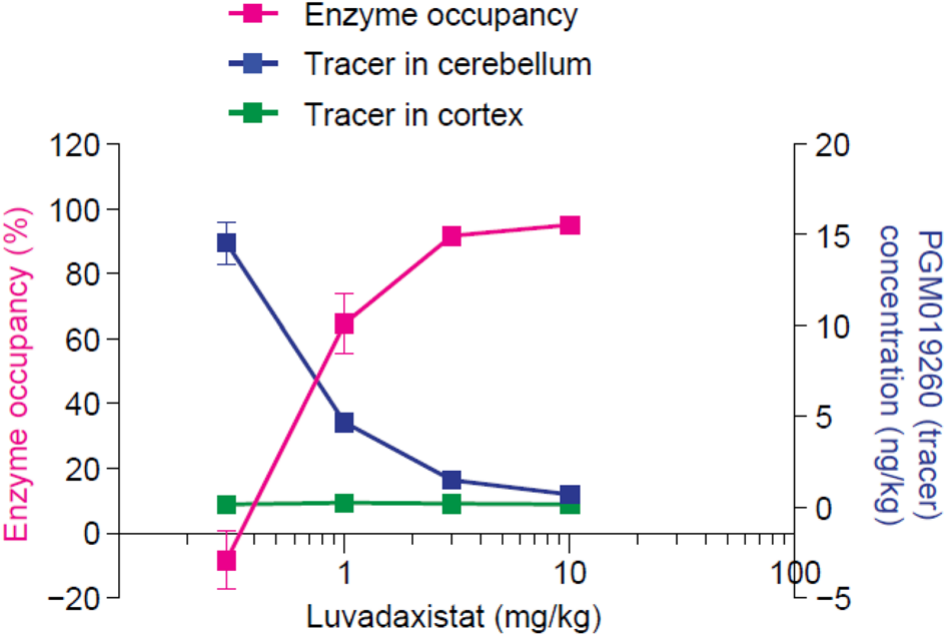
Enzyme occupancy (mean ± SEM, *n* = 4) and tracer regional levels in male C57BL/6 mice pre-treated with luvadaxistat 0.3, 1, 3, and 10 mg/kg p.o. 2 h prior to tracer PGM019260 (60 μg/kg i.v.) resulting in a 50% effective dose (ED_50_) ~ 0.8 mg/kg. i.v., intravenously; p.o., orally; SEM, standard error of the mean

**Fig. 2:**
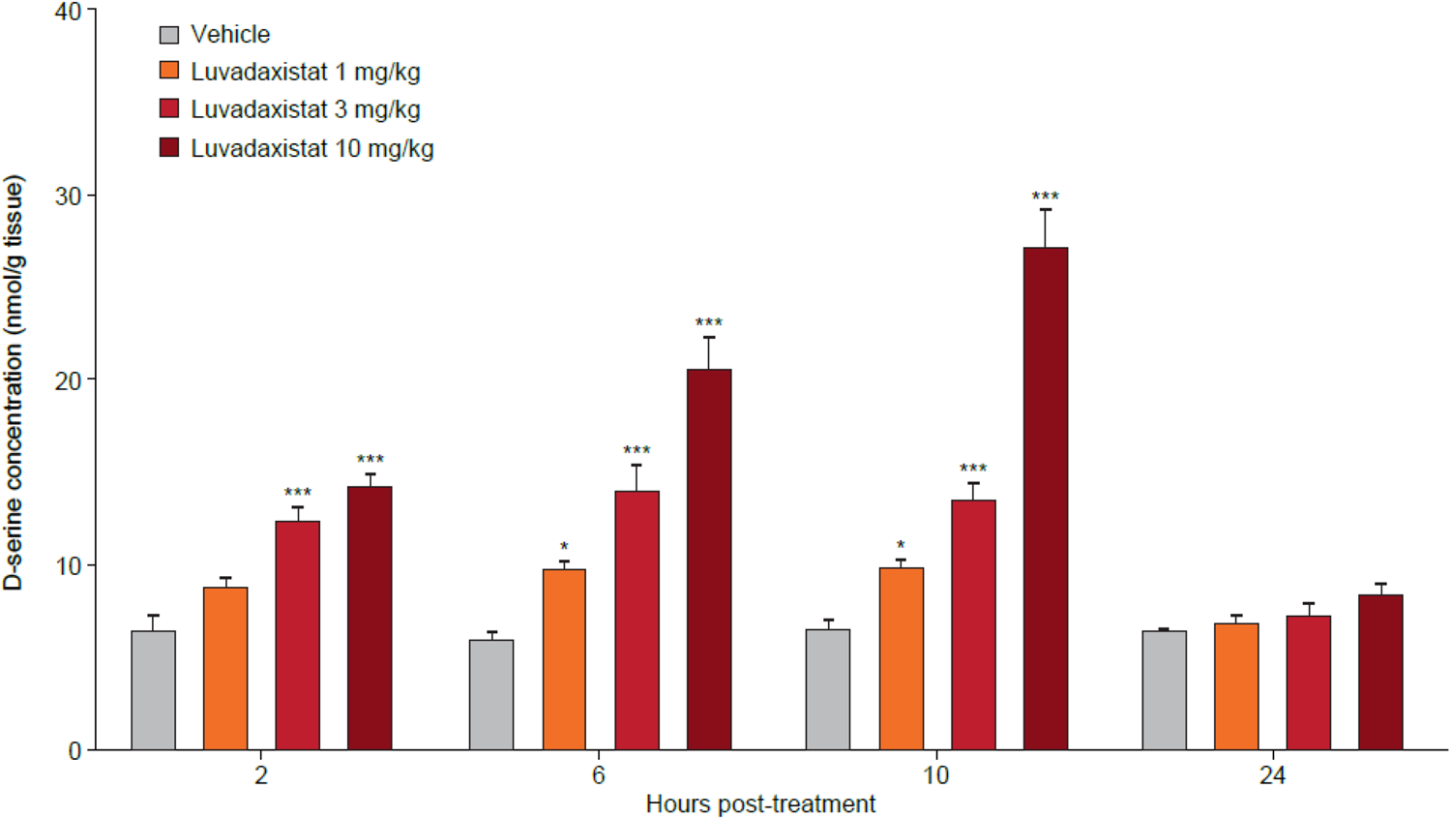
d-Serine levels (mean ± SEM, *n* = 5) in cerebellum of male Wistar rats pre-treated with luvadaxistat 1, 3, and 10 mg/kg p.o. at 2, 6, 10, and 24 h. Data analyzed by two-way ANOVA followed by Bonferroni test. *P < 0.05, ***P < 0.005 compared with the vehicle-treated group. ANOVA, analysis of variance; p.o., orally; SEM, standard error of the mean

**Fig. 3:**
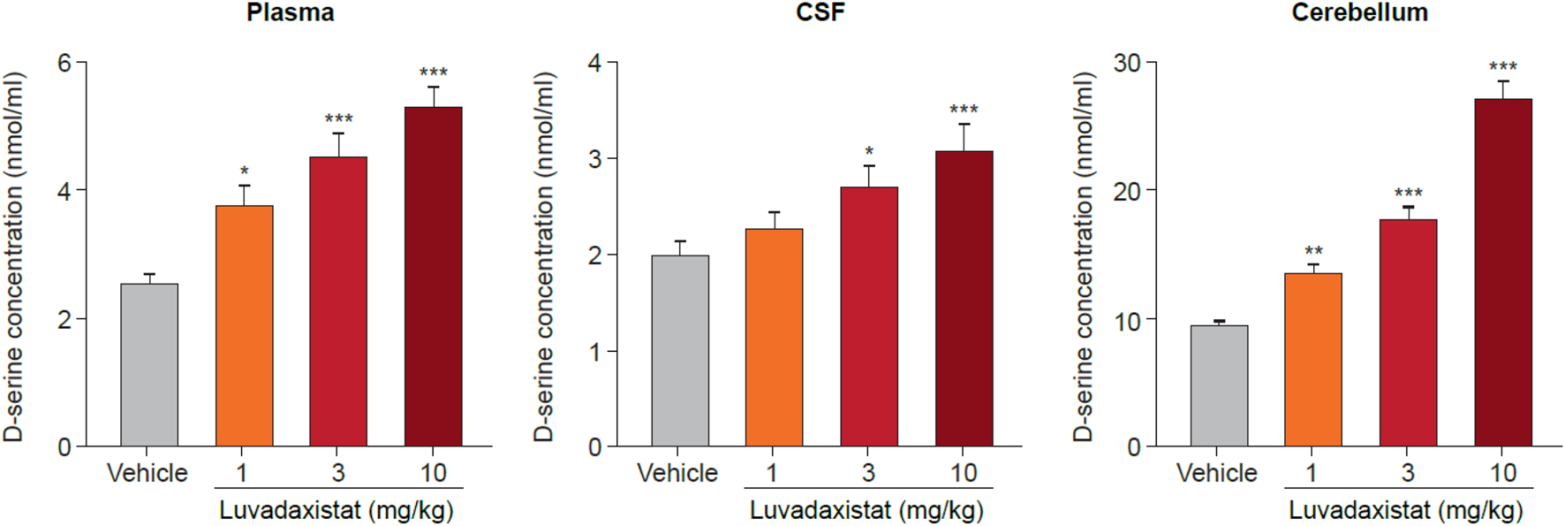
d-Serine levels (mean ± SEM, *n* = 10) in plasma, CSF, and cerebellum of male Wistar rats pre-treated with luvadaxistat 1, 3, and 10 mg/kg p.o. at 6 h. Data analyzed by one-way ANOVA followed by Dunnett’s test. *P < 0.05, **P < 0.01, ***P < 0.001 compared with the vehicle-treated group. ANOVA, analysis of variance; CSF, cerebrospinal fluid; p.o., orally; SEM, standard error of the mean

Enzyme occupancy studies in mice revealed that after 2 h luvadaxistat caused a dose- and exposure-dependent blockade of PGM019260 binding in the cerebellum, with a 50% effective dose (ED_50_) of 0.93 mg/kg (Fig. 1). There was no change in levels of receptor occupancy after chronic dosing of luvadaxistat (data not shown). Inhibition of DAAO by luvadaxistat increased rat cerebellar d-serine levels in a dose- and time-dependent manner with a maximal effect observed at a dose of 10 mg/kg at 10 h post-dose (Fig. 2). This dose-dependent increase in d-serine was also seen in plasma and CSF at 6 h post-dose (Fig. 3). A similar increase in d-serine was also seen in mouse plasma and cerebellum after luvadaxistat inhibition (data not shown). There was no change in the magnitude or time course of d-serine increases after chronic dosing of luvadaxistat (data not shown).

### Effects of Luvadaxistat in Rodent Models of the Cognitive Symptoms of Schizophrenia

#### Episodic Memory

**Fig. 4:**
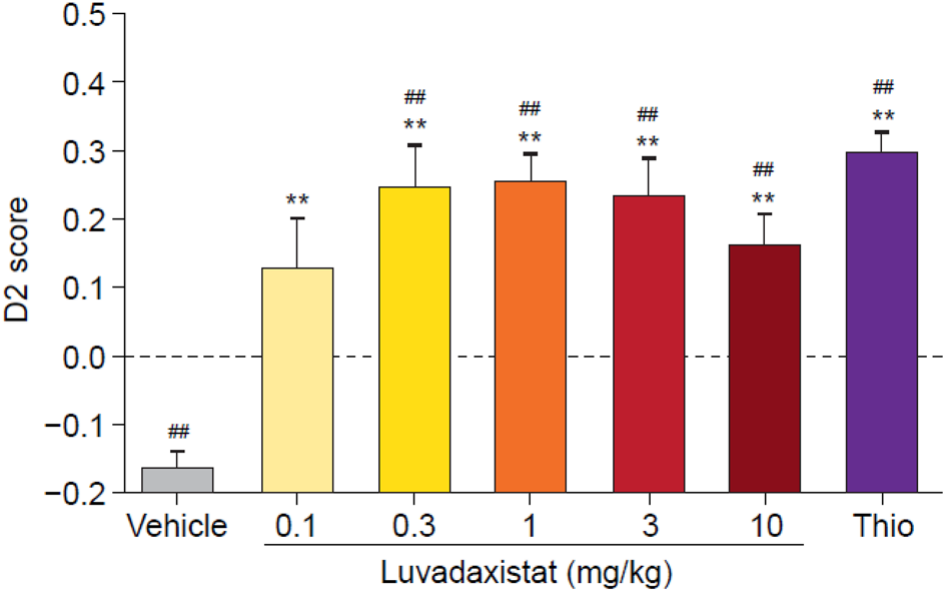
D2 scores (mean ± SEM, *n* = 8) in male C57BL/6J mice pre-treated with luvadaxistat 0.1, 0.3, 1, 3, and 10 mg/kg p.o. or Thio 1.5 mg/kg p.o. Data analyzed by one-way ANOVA followed by Dunnett’s test. **P < 0.01 compared with the vehicle-treated group; ^##^*P* < 0.01 compared with zero (equivalent of not remembering). ANOVA, analysis of variance; D2, discrimination index; NOR, novel object recognition; p.o., orally; SEM, standard error of the mean; Thio, thioperamide

**Fig. 5:**
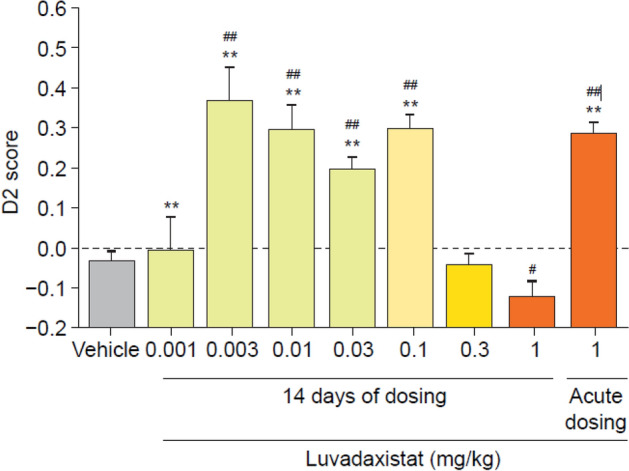
D2 scores (mean ± SEM, *n* = 8) in male C57BL/6J mice pre-treated chronically (14 days) with luvadaxistat 0.001, 0.003, 0.01, 0.03, 0.1, 0.3, and 1 mg/kg p.o. or acutely with luvadaxistat 1 mg/kg p.o. Data analyzed by one-way ANOVA followed by Dunnett’s test. **P < 0.01 compared with the vehicle-treated group; ^#^*P* < 0.05, ^##^*P* < 0.01 compared with zero (equivalent of not remembering). ANOVA, analysis of variance; D2, discrimination index; NOR, novel object recognition; p.o., orally; SEM, standard error of the mean

To evaluate the effects of luvadaxistat on object recognition memory in mice, an inter-trial interval of 3 h in the novel object recognition (NOR) test was introduced. Increasing the inter-trial interval significantly reduced the animals’ ability to identify the novel object, and acute administration of luvadaxistat (doses 0.3–10 mg/kg) significantly reversed this deficit compared with a D2 score of 0 (not remembering) (Fig. 4). The effect of luvadaxistat was comparable to that of the positive control thioperamide at 1.5 mg/kg. Thioperamide is an H3 antagonist that has been shown to enhance arousal and vigilance in rodents [45]. Furthermore, neither luvadaxistat at any dose nor thioperamide significantly affected the T1 or T2 exploration scores. These data suggest that luvadaxistat has the potential to improve time-induced memory deficits. After chronic dosing with luvadaxistat, a leftward shift in the efficacious doses was observed (doses 0.003–0.1 mg/kg) (Fig. 5), with the minimum efficacious dose being reduced and the highest doses no longer efficacious.

### Cognitive Flexibility

We evaluated the effects of acutely and chronically dosed luvadaxistat on sub-chronic PCP-induced deficits in the attentional set-shifting task (ASST) in rats. This test is considered a rodent correlate of the Wisconsin Card Sorting Test in humans, with the extra-dimensional shift (EDS) revealing significant impairments in patients with schizophrenia [40, 41].

**Fig. 6:**
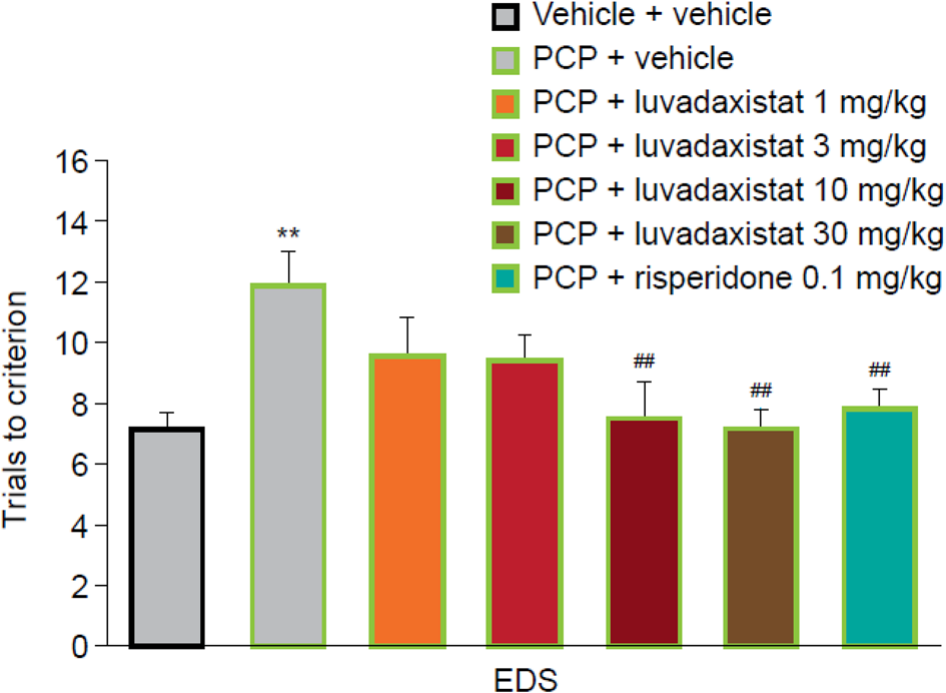
Acute dosing of luvadaxistat in ASST (EDS only shown for ease of reference. Full data set in Supplementary Fig. 1). Trials to criterion (mean ± SEM, *n* = 8–10) in female LH rats pre-treated with luvadaxistat 1, 3, 10, and 30 mg/kg p.o. or risperidone 0.1 mg/kg p.o. Data analyzed by one-way ANOVA followed by Dunnett’s test. **P < 0.01 compared with the vehicle-treated group; ^##^*P* < 0.01 compared with the PCP-treated group. ANOVA, analysis of variance; ASST, attentional set-shifting task; EDS, extra-dimensional shift; LH, Lister hooded; PCP, phencyclidine; p.o., orally; SEM, standard error of the mean

After single dosing of luvadaxistat, a one-way ANOVA followed by post hoc analysis on the EDS phase showed that sub-chronic PCP treatment significantly increased trials to criterion compared with the vehicle-treated group; that is, a robust cognitive deficit was induced by sub-chronic PCP treatment (Fig. 6). The EDS phase deficit was significantly attenuated by acute administration of luvadaxistat at 10 and 30 mg/kg and by 0.1 mg/kg risperidone. This is demonstrated by a significant reduction in trials to criterion compared with the PCP-treated group.

**Fig. 7:**
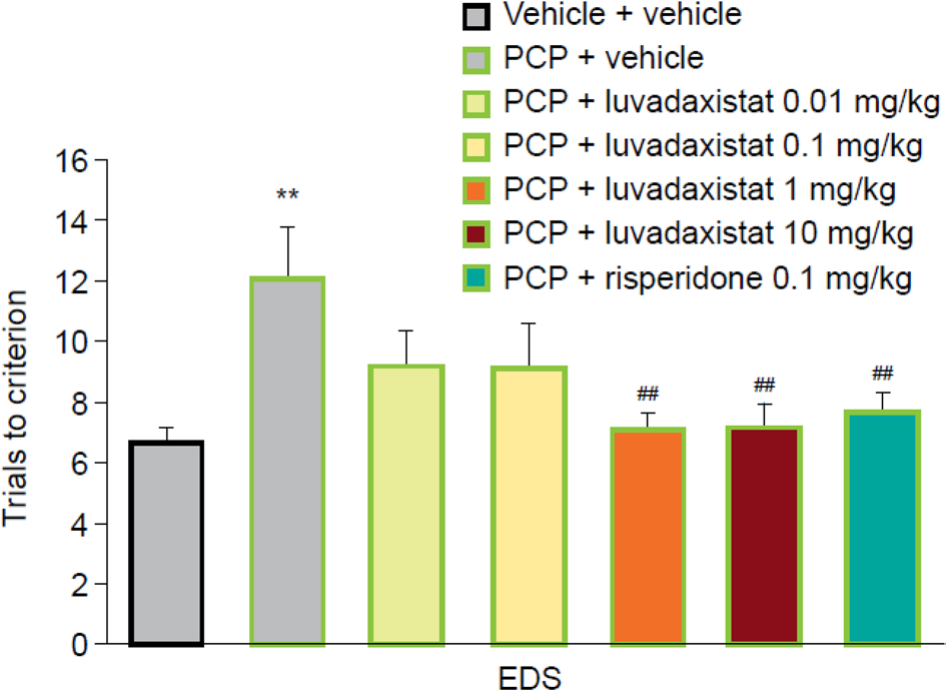
Chronic dosing of luvadaxistat in ASST (EDS only shown for ease of reference. Full data set in Supplementary Fig. 2). Trials to criterion (mean ± SEM, *n* = 8–10) in female LH rats pre-treated chronically (14 days) with luvadaxistat 0.01, 0.1, 1, and 10 mg/kg p.o. or risperidone 0.1 mg/kg p.o. Data analyzed by one-way ANOVA followed by Dunnett’s test. **P < 0.01 compared with the vehicle-treated group; ^#^*P* < 0.05, ^##^*P* < 0.01 compared with the PCP-treated group. ANOVA, analysis of variance; ASST, attentional set-shifting task; EDS, extra-dimensional shift; LH, Lister hooded; PCP, phencyclidine; p.o., orally; SEM, standard error of the mean

After 14-day chronic dosing of luvadaxistat, a one-way ANOVA followed by post hoc analysis on the EDS phase showed that sub-chronic PCP treatment significantly increased trials to criterion compared with the vehicle-treated group; that is, a robust cognitive deficit was induced by sub-chronic PCP treatment (Fig. 7). The EDS phase deficit was significantly attenuated by 14-day administration of luvadaxistat at 1 and 10 mg/kg and by 0.1 mg/kg risperidone. This is demonstrated by a significant reduction in trials to criterion compared with the PCP-treated group.

**Fig. 8:**
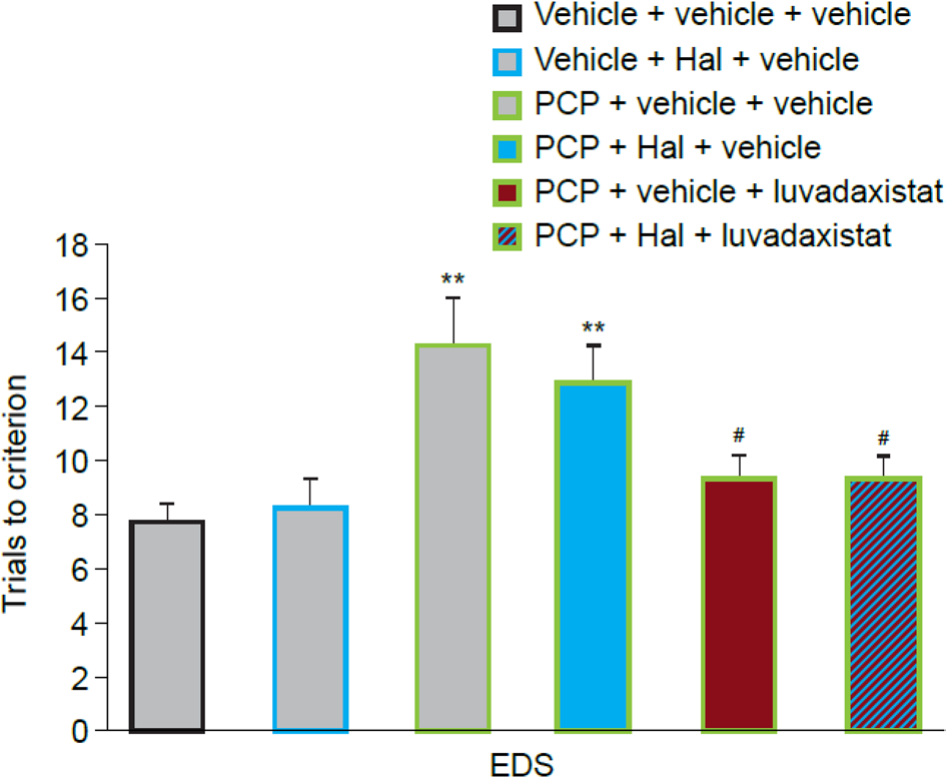
Concurrent dosing of Hal and luvadaxistat in ASST (EDS only shown for ease of reference. Full data set in Supplementary Fig. 3). Trials to criterion (mean ± SEM, *n* = 8–10) in female LH rats pre-treated chronically with luvadaxistat 10 mg/kg p.o. (14 days) and Hal 0.2 mg/kg p.o. (21 days). Data analyzed by one-way ANOVA followed by Dunnett’s test. **P < 0.001 compared with the vehicle + vehicle + vehicle-treated group; ^#^*P* < 0.005 compared with the PCP + vehicle + vehicle-treated group. ANOVA, analysis of variance; ASST, attentional set-shifting task; EDS, extra-dimensional shift; Hal, haloperidol; LH, Lister hooded; PCP, phencyclidine; p.o., orally; SEM, standard error of the mean

After concurrent dosing of haloperidol and luvadaxistat, a one-way ANOVA followed by post hoc analysis on the EDS phase showed that sub-chronic PCP treatment significantly increased trials to criterion compared with the vehicle-treated group; that is, a robust cognitive deficit was induced by sub-chronic PCP treatment (Fig. 8). There was no effect on trials to criterion when 0.2 mg/kg haloperidol was dosed to either vehicle- or PCP-treated animals. The EDS phase deficit was significantly attenuated by 14-day administration of luvadaxistat at 10 mg/kg, either with or without 0.2 mg/kg haloperidol. This is demonstrated by a significant reduction in trials to criterion compared with the PCP-treated group.

Chronic dosing caused a leftward shift in the efficacious doses in this test, without loss of efficacy at the higher doses.

**Fig. 9:**
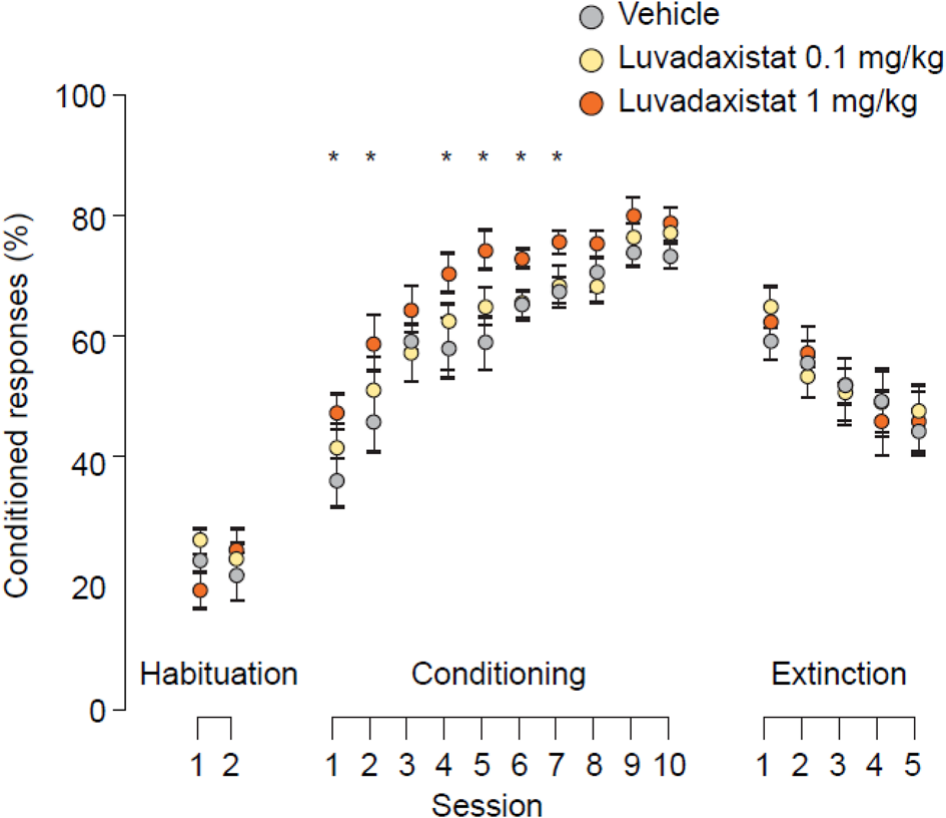
Conditioned responses (mean ± SEM, *n* = 15) in dEBC in male C57Bl6 mice pre-treated chronically (10 days) with luvadaxistat 0.1 and 1 mg/kg p.o. Data analyzed by two-way repeated measures ANOVA followed by Holm–Sidak test for all pairwise multiple comparisons. *P < 0.05 compared with the vehicle-treated group. Asterisk color denotes which group is significantly different from the vehicle-treated group. ANOVA, analysis of variance; dEBC, delay eyeblink conditioning; p.o., orally; SEM, standard error of the mean

**Fig. 10:**
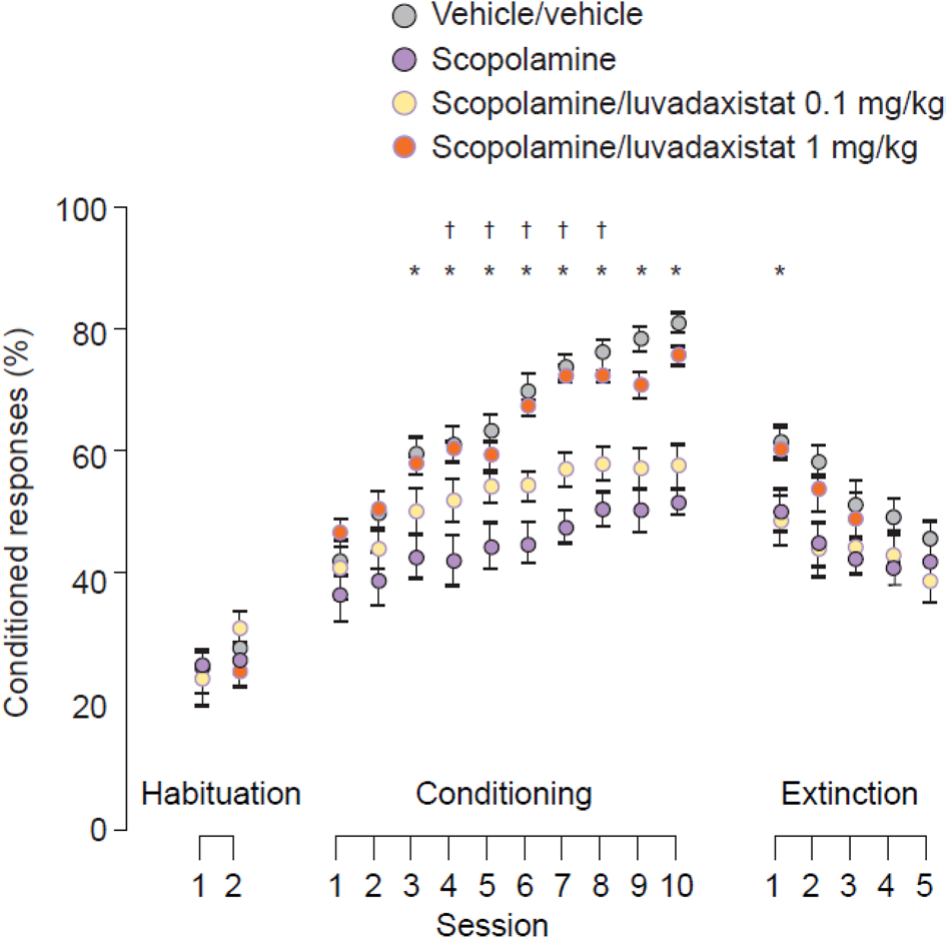
Conditioned responses (mean ± SEM, *n* = 15) in dEBC in male C57Bl6 mice pre-treated chronically (10 days) with luvadaxistat 0.1 and 1 mg/kg p.o. or scopolamine 0.3 mg/kg s.c. Data analyzed by two-way repeated measures ANOVA followed by Holm–Sidak test for all pairwise multiple comparisons. *P < 0.05 compared with the scopolamine-treated group. Asterisk color denotes which group is significantly different from the scopolamine-treated group. ANOVA, analysis of variance; dEBC, delay eyeblink conditioning; p.o., orally; s.c., subcutaneously; SEM, standard error of the mean

To test the impact of luvadaxistat on learning processes mediated by the cerebellum, luvadaxistat was tested in an assay assessing classical conditioning through the use of eyelid responses in dEBC. Luvadaxistat at 1 mg/kg significantly enhanced the acquisition of conditioned responses (Fig. 9) when dosed chronically for 10 days, but this effect was not observed at the lower dose of 0.1 mg/kg. Compared with vehicle-treated controls, 0.3 mg/kg scopolamine significantly decreased the percentage of conditioned responses and luvadaxistat significantly reversed scopolamine-induced deficits when dosed chronically for 10 days at 1 mg/kg and, to a lesser extent, at the lower dose of 0.1 mg/kg (Fig. 10).

### Effects of Luvadaxistat in Rodent Models of Negative Symptoms of Schizophrenia

**Fig. 11:**
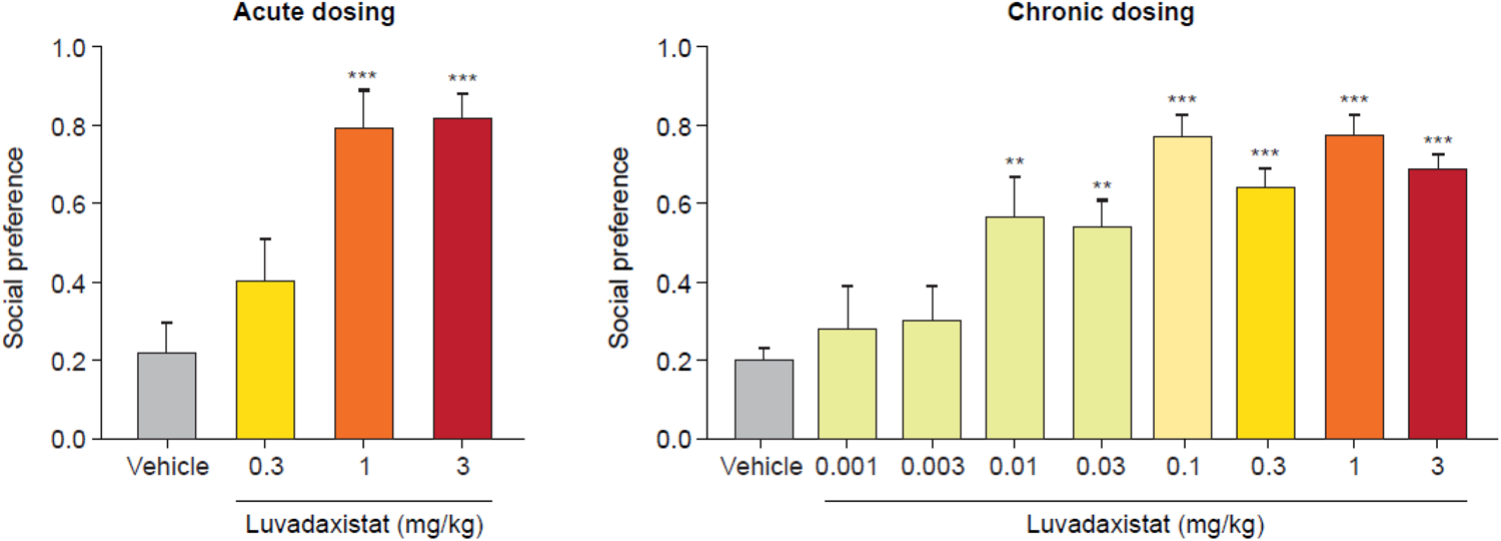
Social preference (mean ± SEM, *n* = 12) in male BALB/c mice pre-treated acutely with luvadaxistat 0.3, 1, and 3 mg/kg p.o. or chronically (14 days) with luvadaxistat 0.001, 0.003, 0.01, 0.03, 0.1, 0.3, 1, and 3 mg/kg p.o. Data analyzed by one-way ANOVA followed by Dunnett’s test. **P < 0.01, ***P < 0.001 compared with the vehicle-treated group. ANOVA, analysis of variance; p.o., orally; SEM, standard error of the mean; SI, social interaction

We explored the ability of luvadaxistat to affect social behavior of juvenile BALB/c mice. Luvadaxistat dose-dependently improved social behavior in the SI test in BALB/c animals when dosed both acutely (1 and 3 mg/kg) and chronically (0.01–3 mg/kg) (Fig. 11).

**Fig. 12:**
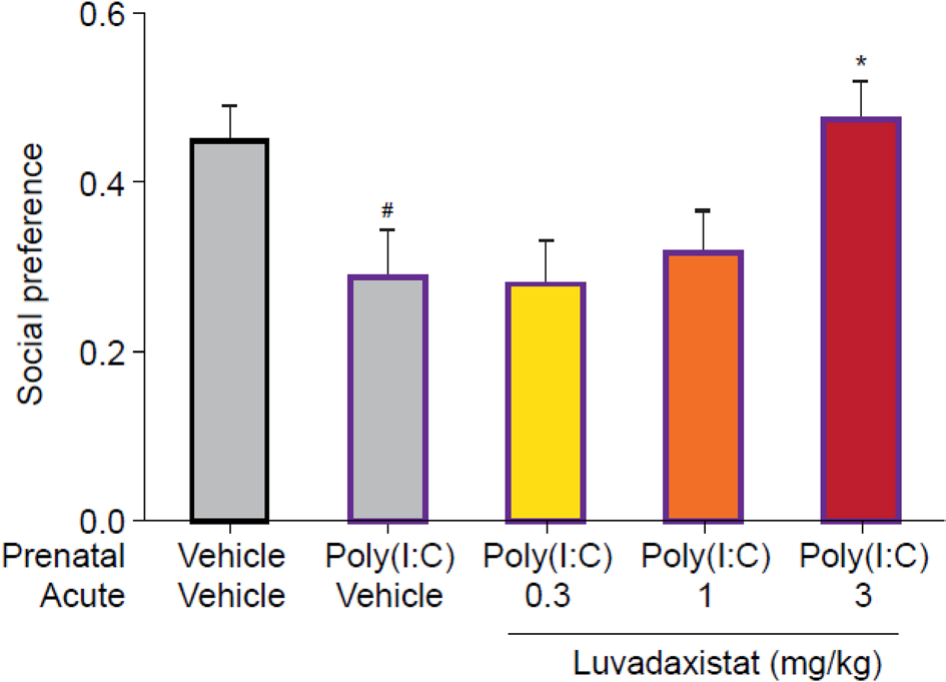
Acute dosing of luvadaxistat significantly reverses a poly(I:C)-induced deficit. Social preference (mean ± SEM, *n* = 12) in male C57BL/6 poly(I:C)-treated mice pre-treated acutely with luvadaxistat 0.3, 1, and 3 mg/kg p.o. Data analyzed by one-way ANOVA followed by Dunnett’s test. *P < 0.05 compared with the poly(I:C):vehicle-treated group; ^#^*P* < 0.05 compared with the vehicle:vehicle-treated group. ANOVA, analysis of variance; p.o., orally; poly(I:C), polyinosinic-polycytidylic acid; SEM, standard error of the mean

We also tested the ability of luvadaxistat to reverse the social impairment seen in mice born from dams treated with poly(I:C), a developmental model of schizophrenia. Luvadaxistat dose-dependently improved SI in these animals when dosed acutely at 3 mg/kg (Fig. 12).

### Effects of Luvadaxistat in Modulation of LTP After In Vivo Dosing

**Fig. 13:**
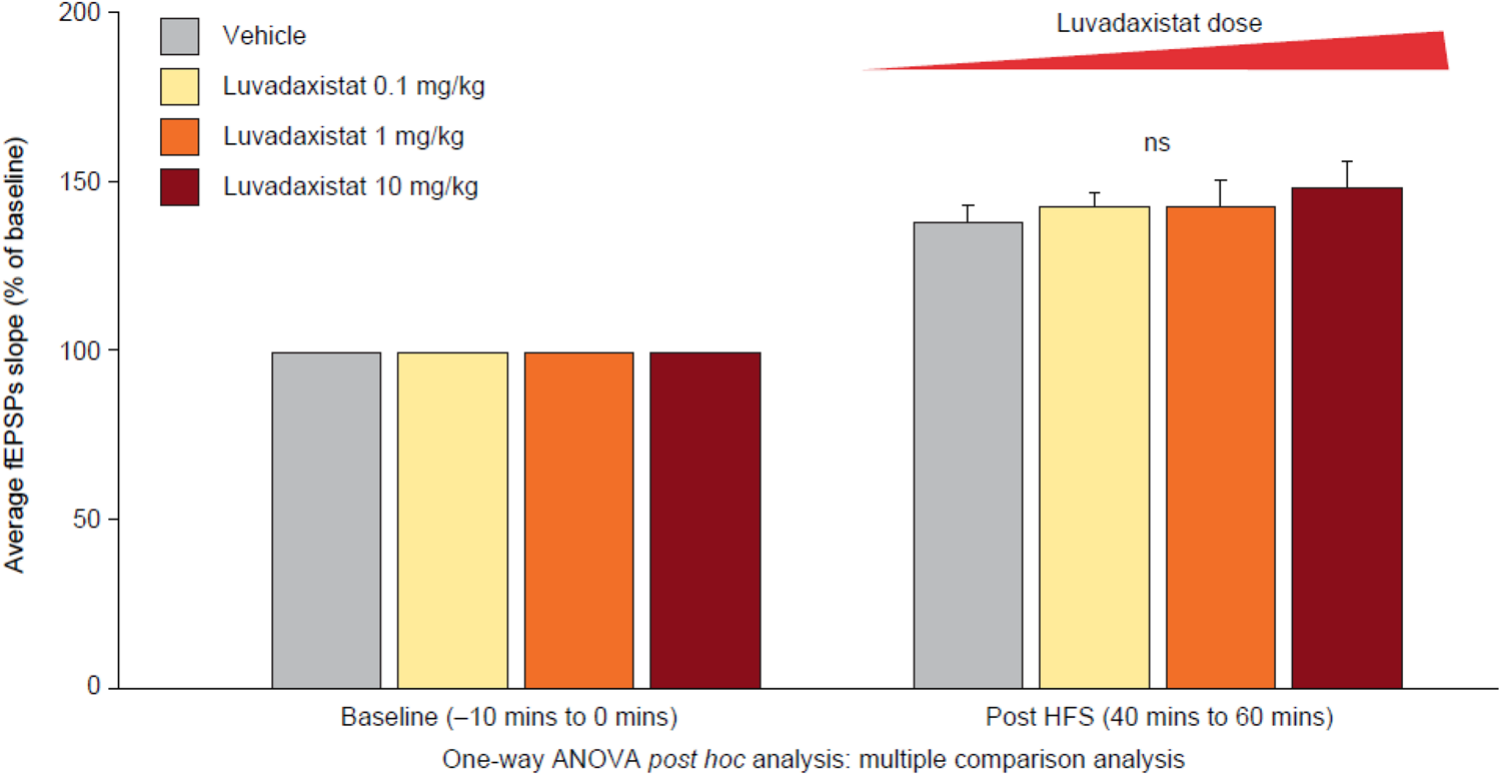
Acute dosing of luvadaxistat did not change LTP measured in hippocampal slices. Columns represent the average slope response during baseline (− 10 to 0 min) and 40–60 min post LTP induction. Time course of the average slopes of elicited field responses following LTP induction by a 10 × TBS stimulation protocol at hippocampal CA1 synapses is shown in Supplementary figures. ANOVA, analysis of variance; fEPSP, field excitatory postsynaptic potential; HFS, high-frequency stimulation; LTP, long-term potentiation; TBS, theta burst stimulation

**Fig. 14:**
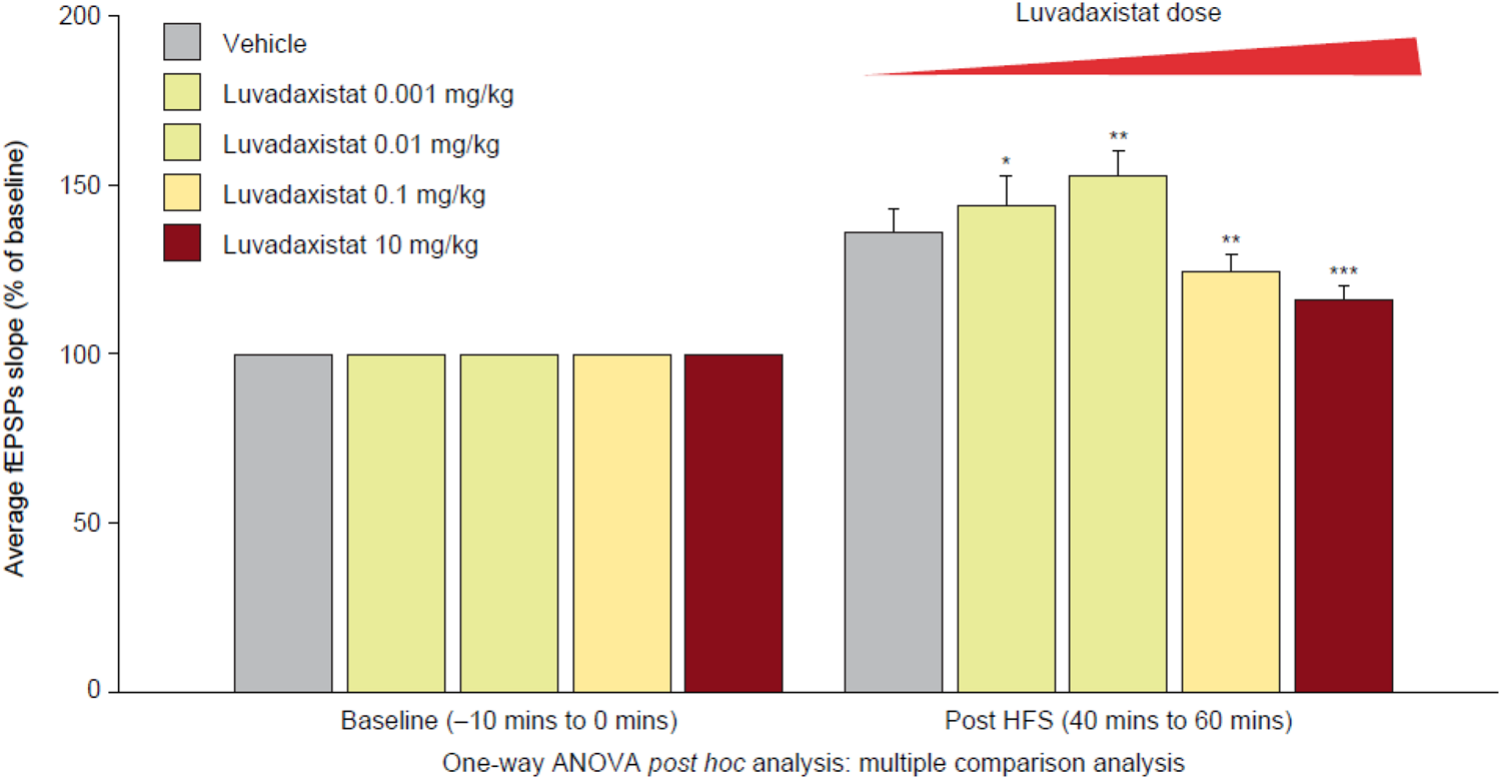
Chronic dosing (14 days; daily) of luvadaxistat induced significant enhancements in LTP when dosed up to 0.01 mg/kg, whereas chronic dosing of luvadaxistat at doses of 0.1 mg/kg and higher induced significant decreases in LTP when compared with vehicle-treated mice. Columns represent the average slope response during baseline (− 10 to 0 min) and 40–60 min post LTP induction. Time course of the average slopes of elicited field responses following LTP induction by a 10 × TBS stimulation protocol at hippocampal CA1 synapses is shown in Supplementary figures. *P < 0.05, **P < 0.01, ***P < 0.001 compared with the vehicle-treated group. ANOVA, analysis of variance; fEPSP, field excitatory postsynaptic potential; HFS, high-frequency stimulation; TBS, theta burst stimulation

To understand if the effects of luvadaxistat and the sensitization phenomenon in the memory performance corresponded to changes in hippocampal plasticity, we carried out ex vivo LTP assessment on hippocampal slices from mice acutely or chronically dosed with luvadaxistat. Single oral doses of luvadaxistat did not significantly affect LTP (based on one-way ANOVA vs. administration of vehicle) (Fig. 13, Supplementary Fig. 4, and Supplementary Fig. 5 for time course details and examples of traces). However, low sub-chronic oral doses of luvadaxistat (0.001 and 0.01 mg/kg) increased LTP, whereas higher doses (0.1 and 10 mg/kg) decreased LTP (all doses *P* < 0.05 vs. vehicle based on a one-way ANOVA) (Fig. 14, Supplementary Fig. 6, and Supplementary Fig. 7 for time course details and examples of traces).

## Discussion

In the present study, we have demonstrated that the investigational drug luvadaxistat is a potent inhibitor of the enzyme DAAO. Through the use of a second, selective DAAO inhibitor PGM19260 used as a tracer compound, we have shown dose-dependent binding of luvadaxistat in the cerebellum. Cerebellum was used as a tissue of choice for the enzyme occupancy studies due to the fact that it is the brain area with the highest expression levels of DAAO [35] for human as well as mouse. Our previous work published in Howley et al. [37] describes the use of the cerebellum to perform enzyme occupancy for DAAO. Building on this work [38], it was found that luvadaxistat causes a dose-dependent increase in d-serine levels following hysteresis at doses causing greater than 70% occupancy at DAAO; a similar effect was seen with other DAAO inhibitor described in Howley et al. [38] and other published reports [46]. It is therefore postulated that enzyme occupancy of DAAO by luvadaxistat causes an increase in d-serine that can be robustly detected in the plasma, CSF, and cerebellum of treated animals after a time period of 5 h. It was for this reason that the majority of studies presented here used a pre-treatment time of 5 h.

To determine the pro-cognitive effects of these increases in d-serine, luvadaxistat was profiled in the NOR test to assess its ability to ameliorate a time-induced memory deficit. Increasing the inter-trial interval has been shown to introduce a challenge to memory performance in rodents and humans [47] and can be used as an alternative to pharmacological intervention such as PCP or ketamine. We have shown that luvadaxistat was able to rescue deficits in memory performance when dosed both acutely or chronically. It was discovered that, after chronic dosing, a leftward shift was seen in the efficacious doses, with a reduction in the minimum efficacious dose and a loss of effect at the highest doses. This was not an isolated phenomenon and was seen on many occasions across multiple tests suggesting that this was not due to simple variability between studies.

Owing to the lack of change in either the d-serine increase in tissues or enzyme occupancy seen after chronic dosing, it was concluded that these effects were not due to accumulation of luvadaxistat, but more likely due to a sensitization effect. This effect may have occurred through changes in neuronal plasticity leading to sensitization within the pathway, while preserving the inverted U-shaped nature of the dose response in accordance with the Yerkes–Dodson law [48]. This sensitization effect was further linked to modulation of neuronal plasticity by showing that after chronic administration of low doses (between 0.001 and 0.01 mg/kg) of luvadaxistat there were significant increases in LTP when measured in hippocampal slices, whereas high doses of luvadaxistat (above 0.1 mg/kg) induced significant decreases in synaptic plasticity.

Inhibition of DAAO enzyme or increasing exogenous levels of d-serine have been shown to modulate synaptic plasticity previously in preclinical studies [49, 50]; findings in humans are also consistent with enhanced synaptic plasticity [51], and it has been shown to be linked to changes in cerebellar as well as forebrain levels of d-serine, which are also known to modulate LTP. The apparently paradoxical finding that doses of luvadaxistat (when dosed chronically) that increase d-serine also inhibit LTP aligns with clinical data that shows an U-shape response in schizophrenia studies [17] with other compounds that also increase glutamatergic tone, such as GlyT1 inhibitors. This suggests that mechanisms designed to increase glutamatergic tone have a very defined dose range for efficacy in cognition-related assessments and that the efficacy of doses outside of this window is clinically difficult to assess.

Luvadaxistat was then profiled in the ASST, a rodent correlate of the human Wisconsin Card Sorting Test [40], and the primate intradimensional/extra-dimensional shift Cambridge Neuropsychological Test Automated Battery [52]. The translatability of this assay can be controversial owing to the fact that atypical antipsychotics are often used as positive controls, even though current treatments are regarded as largely ineffective in treating cognitive symptoms in patients. In this study, the dose of risperidone (0.1 mg/kg) was chosen for its positive effects on cognition probably due to serotonergic mechanisms, particularly antagonism at the 5-hydroxytryptamine receptor 2A [53], rather than an antipsychotic dose, as D2 receptor occupancy would be too low to be clinically meaningful. Again, luvadaxistat was shown to be effective when dosed acutely or chronically and, while there was once more a leftward shift in the efficacious doses, there was no loss of efficacy at the higher dose.

The discrepancy in the sensitization effect seen between the ASST and the NOR test may be due to the fact that the ASST exerts a higher cognitive load than the NOR test. Therefore, the inverted U-shaped dose–response curve was not apparent at the higher doses used in this study, even though changes in neuronal plasticity still led to a sensitization effect. The NOR test, however, places a relatively light cognitive load on the animals; it exploits natural behaviors and requires no conditioning. Therefore, this test may be more sensitive to show downstream plasticity changes that may be masked in more demanding tests such as the ASST.

As any treatment for the cognitive symptoms of schizophrenia would be expected to be used as an adjunctive therapy, it was important to determine whether luvadaxistat would remain efficacious in the presence of the D2 inhibition that is seen with all current antipsychotic treatment. Haloperidol was concurrently dosed with luvadaxistat at a dose predicted to give 70% D2 receptor occupancy and was chosen because it does not show any reversal of ASST deficits on its own. Luvadaxistat reversed the PCP-induced deficit in the EDS to a similar extent in both the presence and absence of haloperidol, suggesting that it would be suitable as an adjunctive therapy.

To investigate the sensitization seen in both the NOR test and the ASST, the effects of chronic dosing of luvadaxistat on d-serine levels in plasma, CSF, and the cerebellum, as well as in vivo receptor occupancy, were determined. However, no changes were seen in any of these studies, suggesting that there is no change at the enzyme protein level or compound exposure/target binding.

DAAO is highly expressed in the cerebellum, and anomalies in cerebellar structure, function, and neurochemistry have been observed in individuals with schizophrenia [54] and in first-degree relatives of those with schizophrenia. Previous findings have also shown an impairment in the cerebellar-dependent associative learning task, dEBC, which may arise from aberrant cerebellar processing. Although other cerebral cortical structures are also involved [55, 56], the cerebellum and its associated circuitry have been reported as essential for this classical conditioning task [57].

To test the ability of luvadaxistat to impact learning processes mediated by the cerebellum, it was tested in a dEBC assay to assess the classical conditioning of eyelid responses. Luvadaxistat enhanced the acquisition of the dEBC when dosed alone, as well as reversing the deficit induced by scopolamine. Luvadaxistat was trialled in a subsequent biomarker study when dosed at 50 and 500 mg for 8 days in 31 patients with schizophrenia [58]. Although there was a trend to enhance cerebellar-dependent learning at the lower dose (something that is in line with the in vivo data, suggesting that low levels of inhibition of the enzyme are required to improve cognition), there was no statistically significant effect seen. The discrepancy between the results seen in the clinical assessment of luvadaxistat compared with those in the preclinical setting are not entirely understood. However, as discussed in O’Donnell et al., a crossover design was used in the clinical study and some task learning may have compromised the capacity to observe changes; whereas that was not a case for the preclinical studies.

d-serine and other inhibitors of DAAO such as sodium benzoate have been shown to affect social and cognitive behavior in humans [32, 35, 59]. To study the ability of luvadaxistat to ameliorate social deficits, we used animal models that reflect some aspects of negative symptoms associated with schizophrenia. We tested luvadaxistat in young BALB/c mice, which have naturally decreased sociability compared with other mouse strains [60], and demonstrated that it induced an increase in sociability in these animals after acute and chronic dosing: it dose-dependently increased time spent with an unfamiliar mouse.

Poly(I:C) triggers an antiviral response in pregnant dams [61], which influences the embryonic development of their progeny. Animals born from these dams show SI deficits starting postnatal day 17. We tested luvadaxistat in the poly(I:C)-induced social deficit model and demonstrated that luvadaxistat induced an increase of sociability in the animals.

In this paper, we have confirmed that inhibition of DAAO by luvadaxistat, probably through a cerebellar mode of action, can ameliorate deficits in rodent models considered to be relevant to schizophrenia. One potential mechanism of action of luvadaxistat is that increases in d-serine levels caused by DAAO inhibition positively impact the activation of NMDARs found on the Purkinje cell–parallel fiber junctions, causing changes in synaptic plasticity [23, 62]. As well as being a potent NMDA-type glutamate receptor co-agonist, d-serine has been shown to be an agonist for the glutamate receptor delta2, which is also implicated in synaptic plasticity, specifically cerebellar long-term depression [23] via downstream alpha-amino-3-hydroxy-5-methyl-4-isoxazolepropionic acid receptor internalization [63]. However, despite increasingly robust evidence for cerebellar involvement in schizophrenia and for the cerebellum as the main site of action of luvadaxistat and other DAAO inhibitors, the link between these molecular effects and the impact on behavior in rodent models or psychiatric symptoms in individuals with schizophrenia is, as yet, poorly understood and an avenue for future exploration.

Our results demonstrate that luvadaxistat is a potent and selective inhibitor of DAAO in vivo and has potential as a promising drug in the treatment of cognitive impairment in schizophrenia. Clinical data from the phase 2 INTERACT study shows that luvadaxistat met its secondary endpoints of cognitive assessment [64]. The current work highlights a remarkable alignment of nonclinical studies (i.e., NOR and cellular LTP), translational studies, and clinical data on predicting and then observing a distinctive inverted U-shaped response on cognitive assays [64, 65]. The primary endpoint in the phase 2 INTERACT study (improvements in adults with negative symptoms of schizophrenia, as measured by the change from baseline on the PANSS NSFS at Day 84), was not met. However, our findings support the potential use of DAAO inhibitors as therapeutic agents to address cognitive symptomatology associated with schizophrenia.

## Supplementary Information

Below is the link to the electronic supplementary material.

Supplementary file1 (DOCX 776 KB)

## Data Availability

The data that support the findings of this study are available from Takeda Pharmaceutical Company Ltd, but restrictions may apply to the availability of these data and so are not publicly available. Data are however available from the authors upon reasonable request and with permission of Takeda Pharmaceutical Company Ltd.

## References

[1] Coyle JT (2012). NMDA receptor and schizophrenia: a brief history. Schizophr Bull.

[2] Yang AC, Tsai SJ (2017). New targets for schizophrenia treatment beyond the dopamine hypothesis. Int J Mol Sci.

[3] Hu W, MacDonald ML, Elswick DE, Sweet RA (2015). The glutamate hypothesis of schizophrenia: evidence from human brain tissue studies. Ann N Y Acad Sci.

[4] Lahti AC, Holcomb HH, Medoff DR, Tamminga CA (1995). Ketamine activates psychosis and alters limbic blood flow in schizophrenia. NeuroReport.

[5] Lahti AC, Koffel B, LaPorte D, Tamminga CA (1995). Subanesthetic doses of ketamine stimulate psychosis in schizophrenia. Neuropsychopharmacology.

[6] Snyder SH (1980). Phencyclidine. Nature.

[7] Hall J, Trent S, Thomas KL, O'Donovan MC, Owen MJ (2015). Genetic risk for schizophrenia: convergence on synaptic pathways involved in plasticity. Biol Psychiatry.

[8] Umbricht D, Alberati D, Martin-Facklam M, Borroni E, Youssef EA, Ostland M, Wallace TL, Knoflach F, Dorflinger E, Wettstein JG, Bausch A, Garibaldi G, Santarelli L (2014). Effect of bitopertin, a glycine reuptake inhibitor, on negative symptoms of schizophrenia: a randomized, double-blind, proof-of-concept study. JAMA Psychiat.

[9] Javitt DC, Zylberman I, Zukin SR, Heresco-Levy U, Lindenmayer JP (1994). Amelioration of negative symptoms in schizophrenia by glycine. Am J Psychiatry.

[10] Traynelis SF, Wollmuth LP, McBain CJ, Menniti FS, Vance KM, Ogden KK, Hansen KB, Yuan H, Myers SJ, Dingledine R (2010). Glutamate receptor ion channels: structure, regulation, and function. Pharmacol Rev.

[11] Balu DT, Coyle JT (2015). The NMDA receptor 'glycine modulatory site' in schizophrenia: D-serine, glycine, and beyond. Curr Opin Pharmacol.

[12] Mothet JP, Parent AT, Wolosker H, Brady RO, Linden DJ, Ferris CD, Rogawski MA, Snyder SH (2000). D-serine is an endogenous ligand for the glycine site of the N-methyl-D-aspartate receptor. Proc Natl Acad Sci USA.

[13] Wolosker H, Balu DT, Coyle JT (2016). The rise and fall of the d-serine-mediated gliotransmission hypothesis. Trends Neurosci.

[14] MacKay MB, Kravtsenyuk M, Thomas R, Mitchell ND, Dursun SM, Baker GB (2019). D-Serine: potential therapeutic agent and/or biomarker in schizophrenia and depression?. Front Psychiatry.

[15] Choi DW, Koh JY, Peters S (1988). Pharmacology of glutamate neurotoxicity in cortical cell culture: attenuation by NMDA antagonists. J Neurosci.

[16] Harvey RJ, Yee BK (2013). Glycine transporters as novel therapeutic targets in schizophrenia, alcohol dependence and pain. Nat Rev Drug Discov.

[17] Dunayevich E, Buchanan RW, Chen CY, Yang J, Nilsen J, Dietrich JM, Sun H, Marder S (2017). Efficacy and safety of the glycine transporter type-1 inhibitor AMG 747 for the treatment of negative symptoms associated with schizophrenia. Schizophr Res.

[18] Bugarski-Kirola D, Blaettler T, Arango C, Fleischhacker WW, Garibaldi G, Wang A, Dixon M, Bressan RA, Nasrallah H, Lawrie S, Napieralski J, Ochi-Lohmann T, Reid C, Marder SR (2017). Bitopertin in negative symptoms of schizophrenia-results from the phase III FlashLyte and DayLyte studies. Biol Psychiatry.

[19] Matsui T, Sekiguchi M, Hashimoto A, Tomita U, Nishikawa T, Wada K (1995). Functional comparison of D-serine and glycine in rodents: the effect on cloned NMDA receptors and the extracellular concentration. J Neurochem.

[20] Paoletti P, Bellone C, Zhou Q (2013). NMDA receptor subunit diversity: impact on receptor properties, synaptic plasticity and disease. Nat Rev Neurosci.

[21] Madry C, Mesic I, Betz H, Laube B (2007). The N-terminal domains of both NR1 and NR2 subunits determine allosteric Zn2+ inhibition and glycine affinity of N-methyl-D-aspartate receptors. Mol Pharmacol.

[22] Papouin T, Ladepeche L, Ruel J, Sacchi S, Labasque M, Hanini M, Groc L, Pollegioni L, Mothet JP, Oliet SH (2012). Synaptic and extrasynaptic NMDA receptors are gated by different endogenous coagonists. Cell.

[23] Kakegawa W, Miyoshi Y, Hamase K, Matsuda S, Matsuda K, Kohda K, Emi K, Motohashi J, Konno R, Zaitsu K, Yuzaki M (2011). D-serine regulates cerebellar LTD and motor coordination through the delta2 glutamate receptor. Nat Neurosci.

[24] Kantrowitz JT, Epstein ML, Lee M, Lehrfeld N, Nolan KA, Shope C, Petkova E, Silipo G, Javitt DC (2018). Improvement in mismatch negativity generation during d-serine treatment in schizophrenia: correlation with symptoms. Schizophr Res.

[25] Hashimoto K, Fukushima T, Shimizu E, Komatsu N, Watanabe H, Shinoda N, Nakazato M, Kumakiri C, Okada S, Hasegawa H, Imai K, Iyo M (2003). Decreased serum levels of D-serine in patients with schizophrenia: evidence in support of the N-methyl-D-aspartate receptor hypofunction hypothesis of schizophrenia. Arch Gen Psychiatry.

[26] Hashimoto K, Engberg G, Shimizu E, Nordin C, Lindstrom LH, Iyo M (2005). Reduced D-serine to total serine ratio in the cerebrospinal fluid of drug naive schizophrenic patients. Prog Neuropsychopharmacol Biol Psychiatry.

[27] Hashimoto K, Bruno D, Nierenberg J, Marmar CR, Zetterberg H, Blennow K, Pomara N (2016). Abnormality in glutamine-glutamate cycle in the cerebrospinal fluid of cognitively intact elderly individuals with major depressive disorder: a 3-year follow-up study. Transl Psychiatry.

[28] Bendikov I, Nadri C, Amar S, Panizzutti R, De Miranda J, Wolosker H, Agam G (2007). A CSF and postmortem brain study of D-serine metabolic parameters in schizophrenia. Schizophr Res.

[29] Ohnuma T, Sakai Y, Maeshima H, Hatano T, Hanzawa R, Abe S, Kida S, Shibata N, Suzuki T, Arai H (2008). Changes in plasma glycine, L-serine, and D-serine levels in patients with schizophrenia as their clinical symptoms improve: results from the Juntendo University Schizophrenia Projects (JUSP). Prog Neuropsychopharmacol Biol Psychiatry.

[30] Calcia MA, Madeira C, Alheira FV, Silva TC, Tannos FM, Vargas-Lopes C, Goldenstein N, Brasil MA, Ferreira ST, Panizzutti R (2012). Plasma levels of D-serine in Brazilian individuals with schizophrenia. Schizophr Res.

[31] Kantrowitz JT, Malhotra AK, Cornblatt B, Silipo G, Balla A, Suckow RF, D'Souza C, Saksa J, Woods SW, Javitt DC (2010). High dose D-serine in the treatment of schizophrenia. Schizophr Res.

[32] Kantrowitz JT, Woods SW, Petkova E, Cornblatt B, Corcoran CM, Chen H, Silipo G, Javitt DC (2015). D-serine for the treatment of negative symptoms in individuals at clinical high risk of schizophrenia: a pilot, double-blind, placebo-controlled, randomised parallel group mechanistic proof-of-concept trial. Lancet Psychiatry.

[33] Tsai G, Yang P, Chung LC, Lange N, Coyle JT (1998). D-serine added to antipsychotics for the treatment of schizophrenia. Biol Psychiatry.

[34] Hasegawa H, Masuda N, Natori H, Shinohara Y, Ichida K (2019). Pharmacokinetics and toxicokinetics of d-serine in rats. J Pharm Biomed Anal.

[35] Lin CH, Yang HT, Chen PK, Wang SH, Lane HY (2020). Precision medicine of sodium benzoate for the treatment of behavioral and psychological symptoms of dementia (BPSD). Neuropsychiatr Dis Treat.

[36] Almond SL, Fradley RL, Armstrong EJ, Heavens RB, Rutter AR, Newman RJ, Chiu CS, Konno R, Hutson PH, Brandon NJ (2006). Behavioral and biochemical characterization of a mutant mouse strain lacking D-amino acid oxidase activity and its implications for schizophrenia. Mol Cell Neurosci.

[37] Yoneyama T, Sato S, Sykes A, Fradley R, Stafford S, Bechar S, Howley E, Patel T, Tagawa Y, Moriwaki T, Asahi S (2020). Mechanistic multilayer quantitative model for nonlinear pharmacokinetics, target occupancy and pharmacodynamics (PK/TO/PD) relationship of D-amino acid oxidase inhibitor, TAK-831 in mice. Pharm Res.

[38] Howley E, Bestwick M, Fradley R, Harrison H, Leveridge M, Okada K, Fieldhouse C, Farnaby W, Canning H, Sykes AP, Merchant K, Hazel K, Kerr C, Kinsella N, Walsh L, Livermore DG, Hoffman I, Ellery J, Mitchell P, Patel T, Carlton M, Barnes M, Miller DJ (2017). Assessment of the target engagement and D-serine biomarker profiles of the D-amino acid oxidase inhibitors sodium benzoate and PGM030756. Neurochem Res.

[39] Brandish PE, Chiu CS, Schneeweis J, Brandon NJ, Leech CL, Kornienko O, Scolnick EM, Strulovici B, Zheng W (2006). A cell-based ultra-high-throughput screening assay for identifying inhibitors of D-amino acid oxidase. J Biomol Screen.

[40] McLean SL, Beck JP, Woolley ML, Neill JC (2008). A preliminary investigation into the effects of antipsychotics on sub-chronic phencyclidine-induced deficits in attentional set-shifting in female rats. Behav Brain Res.

[41] McLean SL, Idris NF, Grayson B, Gendle DF, Mackie C, Lesage AS, Pemberton DJ, Neill JC (2012). PNU-120596, a positive allosteric modulator of alpha7 nicotinic acetylcholine receptors, reverses a sub-chronic phencyclidine-induced cognitive deficit in the attentional set-shifting task in female rats. J Psychopharmacol.

[42] Neill JC, Barnes S, Cook S, Grayson B, Idris NF, McLean SL, Snigdha S, Rajagopal L, Harte MK (2010). Animal models of cognitive dysfunction and negative symptoms of schizophrenia: focus on NMDA receptor antagonism. Pharmacol Ther.

[43] Gruart A, Munoz MD, Delgado-Garcia JM (2006). Involvement of the CA3-CA1 synapse in the acquisition of associative learning in behaving mice. J Neurosci.

[44] Okamura H, Yasugaki S, Suzuki-Abe H, Arai Y, Sakurai K, Yanagisawa M, Takizawa H, Hayashi Y (2022). Long-term effects of repeated social defeat stress on brain activity during social interaction in BALB/c mice. eNeuro.

[45] Pascoli V, Boer-Saccomani C, Hermant JF (2009). H3 receptor antagonists reverse delay-dependent deficits in novel object discrimination by enhancing retrieval. Psychopharmacology.

[46] Strick CA, Li C, Scott L, Harvey B, Hajos M, Steyn SJ, Piotrowski MA, James LC, Downs JT, Rago B, Becker SL, El-Kattan A, Xu Y, Ganong AH, Tingley FD, Ramirez AD, Seymour PA, Guanowsky V, Majchrzak MJ, Fox CB, Schmidt CJ, Duplantier AJ (2011). Modulation of NMDA receptor function by inhibition of D-amino acid oxidase in rodent brain. Neuropharmacology.

[47] Leger M, Quiedeville A, Bouet V, Haelewyn B, Boulouard M, Schumann-Bard P, Freret T (2013). Object recognition test in mice. Nat Protoc.

[48] Mair RG, Onos KD, Hembrook JR (2011). Cognitive activation by central thalamic stimulation: the yerkes-dodson law revisited. Dose Response.

[49] Zhang Z, Gong N, Wang W, Xu L, Xu TL (2008). Bell-shaped D-serine actions on hippocampal long-term depression and spatial memory retrieval. Cereb Cortex.

[50] Hopkins SC, Campbell UC, Heffernan ML, Spear KL, Jeggo RD, Spanswick DC, Varney MA, Large TH (2013). Effects of D-amino acid oxidase inhibition on memory performance and long-term potentiation in vivo. Pharmacol Res Perspect.

[51] Kantrowitz JT, Epstein ML, Beggel O, Rohrig S, Lehrfeld JM, Revheim N, Lehrfeld NP, Reep J, Parker E, Silipo G, Ahissar M, Javitt DC (2016). Neurophysiological mechanisms of cortical plasticity impairments in schizophrenia and modulation by the NMDA receptor agonist D-serine. Brain.

[52] Tyson PJ, Laws KR, Roberts KH, Mortimer AM (2004). Stability of set-shifting and planning abilities in patients with schizophrenia. Psychiatry Res.

[53] Neill JC, Harte MK, Haddad PM, Lydall ES, Dwyer DM (2014). Acute and chronic effects of NMDA receptor antagonists in rodents, relevance to negative symptoms of schizophrenia: a translational link to humans. Eur Neuropsychopharmacol.

[54] Bolbecker AR, Kent JS, Petersen IT, Klaunig MJ, Forsyth JK, Howell JM, Westfall DR, O'Donnell BF, Hetrick WP (2014). Impaired cerebellar-dependent eyeblink conditioning in first-degree relatives of individuals with schizophrenia. Schizophr Bull.

[55] Sanchez-Campusano R, Gruart A, Delgado-Garcia JM (2009). Dynamic associations in the cerebellar-motoneuron network during motor learning. J Neurosci.

[56] Hasan MT, Hernandez-Gonzalez S, Dogbevia G, Trevino M, Bertocchi I, Gruart A, Delgado-Garcia JM (2013). Role of motor cortex NMDA receptors in learning-dependent synaptic plasticity of behaving mice. Nat Commun.

[57] Christian KM, Thompson RF (2003). Neural substrates of eyeblink conditioning: acquisition and retention. Learn Mem.

[58] O'Donnell P, Dong C, Murthy V, Asgharnejad M, Du X, Summerfelt A, Lu H, Xu L, Wendland JR, Dunayevich E, Buhl DL, Litman R, Hetrick WP, Hong LE, Rosen LB (2023). The D-amino acid oxidase inhibitor luvadaxistat improves mismatch negativity in patients with schizophrenia in a randomized trial. Neuropsychopharmacology.

[59] Lin CH, Chen PK, Chang YC, Chuo LJ, Chen YS, Tsai GE, Lane HY (2014). Benzoate, a D-amino acid oxidase inhibitor, for the treatment of early-phase Alzheimer disease: a randomized, double-blind, placebo-controlled trial. Biol Psychiatry.

[60] Brodkin ES (2007). BALB/c mice: low sociability and other phenotypes that may be relevant to autism. Behav Brain Res.

[61] Missault S, Van den Eynde K, Vanden Berghe W, Fransen E, Weeren A, Timmermans JP, Kumar-Singh S, Dedeurwaerdere S (2014). The risk for behavioural deficits is determined by the maternal immune response to prenatal immune challenge in a neurodevelopmental model. Brain Behav Immun.

[62] D'Ascenzo M, Podda MV, Grassi C (2014). The role of D-serine as co-agonist of NMDA receptors in the nucleus accumbens: relevance to cocaine addiction. Front Synaptic Neurosci.

[63] Hirai H, Launey T, Mikawa S, Torashima T, Yanagihara D, Kasaura T, Miyamoto A, Yuzaki M (2003). New role of delta2-glutamate receptors in AMPA receptor trafficking and cerebellar function. Nat Neurosci.

[64] Murthy V, Hanson E, DeMartinis N, Asgharnejad M, Dong C, Evans R, Ge T, Dunayevich E, Singh J, Ratti E, Galderisi S (2021). Luvadaxistat, an investigational D-amino acid oxidase inhibitor, was associated with signals of efficacy in cognitive impairment associated with schizophrenia but not negative symptoms: results from the interact study. Neuropsychopharmacology.

[65] O’Donnell P, Dong C, Murthy V, Asgharnejad M, Du X, Summerfelt A, Wendland J, Dunayevich E, Litman R, Hetrick W, Hong E, Rosen L (2021). Luvadaxistat, a D-amino acid oxidase inhibitor, improves mismatch negativity in patients with schizophrenia. Neuropsychopharmacology.

