# Correction to: Luvadaxistat: A Novel Potent and Selective d-Amino Acid Oxidase Inhibitor Improves Cognitive and Social Deficits in Rodent Models for Schizophrenia

**DOI:** 10.1007/s11064-023-03987-9

**Published:** 2023-07-13

**Authors:** Rosa Fradley, Pascal Goetghebeur, David Miller, Russell Burley, Sarah Almond, Agnès Gruart i Massó, José M. Delgado García, Bin Zhu, Eimear Howley, Jo C. Neill, Ben Grayson, Philip Gaskin, Mark Carlton, Ian Gray, Jordi Serrats, Ceri H. Davies

**Affiliations:** 1 Neuroscience Drug Discovery Unit, Takeda, Cambridge, UK; 2 Division of Neurosciences, Pablo de Olavide University, Seville, Spain; 3 Division of Pharmacy and Optometry, School of Health Sciences, University of Manchester, Manchester, UK; 4 Neuroscience Drug Discovery Unit, Takeda California, 9625 Towne Centre Dr, San Diego, CA 92121 USA; 5 Takeda Pharmaceuticals Company Limited, Fujisawa, Kanagawa Japan

## Correction to: Neurochemical Research 10.1007/s11064-023-03956-2

The article ‘‘Luvadaxistat: A Novel Potent and Selective d‑Amino Acid Oxidase Inhibitor Improves Cognitive and Social Defcits in Rodent Models for Schizophrenia’’, written by Rosa Fradley, Pascal Goetghebeur, David Miller, Russell Burley, Sarah Almond, Agnès Gruart i Massó, José M. Delgado García, Bin Zhu, Eimear Howley, Jo C. Neill, Ben Grayson, Philip Gaskin, Mark Carlton, Ian Gray, Jordi Serrats, and Ceri H. Davies, was originally published electronically on the publisher’s internet portal (https://link.springer.com/article/10.1007/s11064-023-03956-2) on 8 June 2023 without open access. With the author(s)’ decision to opt for Open Choice, the copyright of the article changed on 27 June 2023 to “The Author(s) 2023” and the article is forthwith distributed under the terms of the Creative Commons Attribution Section 4.1 International License (http://creativecommons.org/licenses/by/4.0/), which permits use, duplication, adaptation, distribution and reproduction in any medium or format, as long as you give appropriate credit to the original author(s) and the source, provide a link to the Creative Commons license and indicate if changes were made.

The original article has been corrected.

