# Supplementary material for: Luvadaxistat: A Novel Potent and Selective d-Amino Acid Oxidase Inhibitor Improves Cognitive and Social Deficits in Rodent Models for Schizophrenia
Source: Neurochem Res. 2023 Jun 8;48(10):3027–41. doi: 10.1007/s11064-023-03956-2 (PMC10471729; doi:10.1007/s11064-023-03956-2)

**Supplementary Information**

**Luvadaxistat: A Novel Potent and Selective D-Amino Acid Oxidase Inhibitor Improves Cognitive and Social Deficits in Rodent Models for Schizophrenia**

*Neurochemical Research*

Rosa Fradley • Pascal Goetghebeur • David Miller • Russell Burley • Sarah Almond • Agnès Gruart i Massó • José M. Delgado García • Bin Zhu • Eimear Howley • Jo C. Neill • Ben Grayson • Philip Gaskin • Mark Carlton • Ian Gray • Jordi Serrats • Ceri H. Davies

**Corresponding Author**

Jordi Serrats, Neuroscience Drug Discovery Unit, Takeda, Cambridge, UK (at the time the study was conducted)

E-mail: [jordi@engrail.com](mailto:jordi@engrail.com)

**Supplementary Fig. 1** Acute dosing of luvadaxistat significantly attenuates a PCP-induced deficit in ASST. Trials to criterion (mean±SEM, n=8–10) in female LH rats pre-treated with luvadaxistat 1, 3, 10, and 30 mg/kg p.o. and risperidone 0.1 mg/kg p.o. Data from this study are presented as mean trials to criterion and analyzed by a repeated measures two-way ANOVA using phase as a within-subjects factor and drug treatment as a between-subjects factor. Where a significant effect was detected, a one-way ANOVA was then performed followed by a Dunnett’s post hoc *t*-test in order to compare treatment groups versus the appropriate control. *P<0.05, **P<0.01, ***P<0.005 compared with the vehicle-treated group; ^#^P<0.05, ^##^P<0.01, ^###^P<0.005 compared with the PCP-treated group. ANOVA, analysis of variance; ASST, attentional set-shifting task; CD, compound discrimination; EDS, extra-dimensional shift; IDS, intra-dimensional shift; LH, Lister hooded; PCP, phencyclidine; p.o., orally; Rev, reversal; SD, simple discrimination; SEM, standard error of the mean


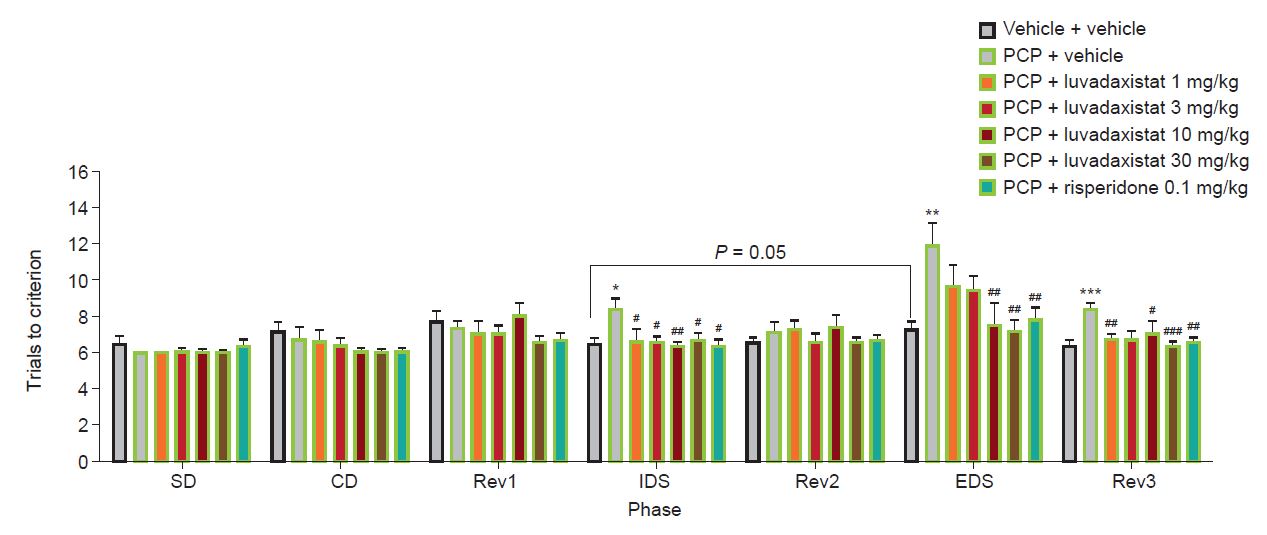


**Supplementary Fig. 2** Chronic dosing of luvadaxistat significantly attenuates a PCP-induced deficit in ASST. Trials to criterion (mean±SEM, n=8–10) in female LH rats pre-treated chronically (14 days) with luvadaxistat 0.01, 0.1, 1, and 10 mg/kg p.o. and risperidone 0.1 mg/kg p.o. Data from this study are presented as mean trials to criterion and analyzed by a repeated measures two-way ANOVA using phase as a within-subjects factor and drug treatment as a between-subjects factor. Where a significant effect was detected, a one-way ANOVA was then performed followed by a Dunnett’s post hoc *t*-test in order to compare treatment groups versus the appropriate control. **P<0.01 compared with the vehicle-treated group; ^#^P<0.05, ^##^P<0.01 compared with the PCP-treated group. ANOVA, analysis of variance; ASST, attentional set-shifting task; CD, compound discrimination; EDS, extra-dimensional shift; IDS, intra-dimensional shift; LH, Lister hooded; PCP, phencyclidine; p.o., orally; Rev, reversal; SD, simple discrimination; SEM, standard error of the mean


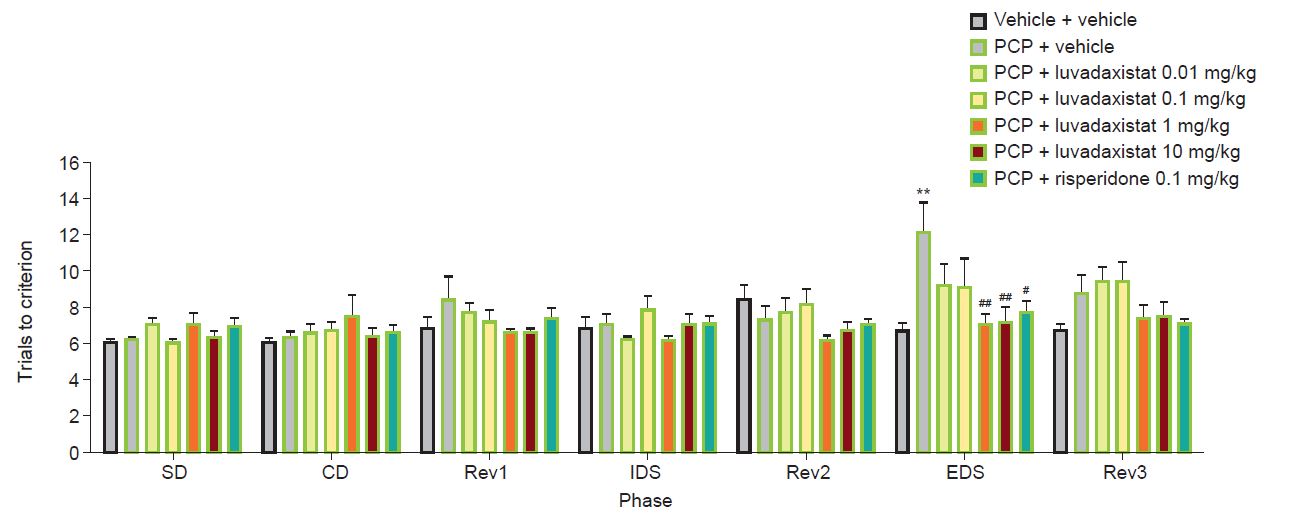


**Supplementary Fig. 3** Concurrent dosing of Hal and luvadaxistat significantly attenuates a PCP-induced deficit in ASST. Trials to criterion (mean±SEM, n=8–10) in female LH rats pre-treated chronically with luvadaxistat 10 mg/kg p.o. (14 days) and Hal 0.2 mg/kg p.o. (21 days). Data from this study are presented as mean trials to criterion and analyzed by a repeated measures two-way ANOVA using phase as a within-subjects factor and drug treatment as a between-subjects factor. Where a significant effect was detected, a one-way ANOVA was then performed followed by a Dunnett’s post hoc *t*-test in order to compare treatment groups versus the appropriate control. **P<0.001 compared with the vehicle-treated group; ^#^P<0.05 compared with the PCP-treated group. ANOVA, analysis of variance; ASST, attentional set-shifting task; CD, compound discrimination; EDS, extra-dimensional shift; Hal, haloperidol; IDS, intra-dimensional shift; LH, Lister hooded; PCP, phencyclidine; p.o., orally; Rev, reversal; SD, simple discrimination; SEM, standard error of the mean


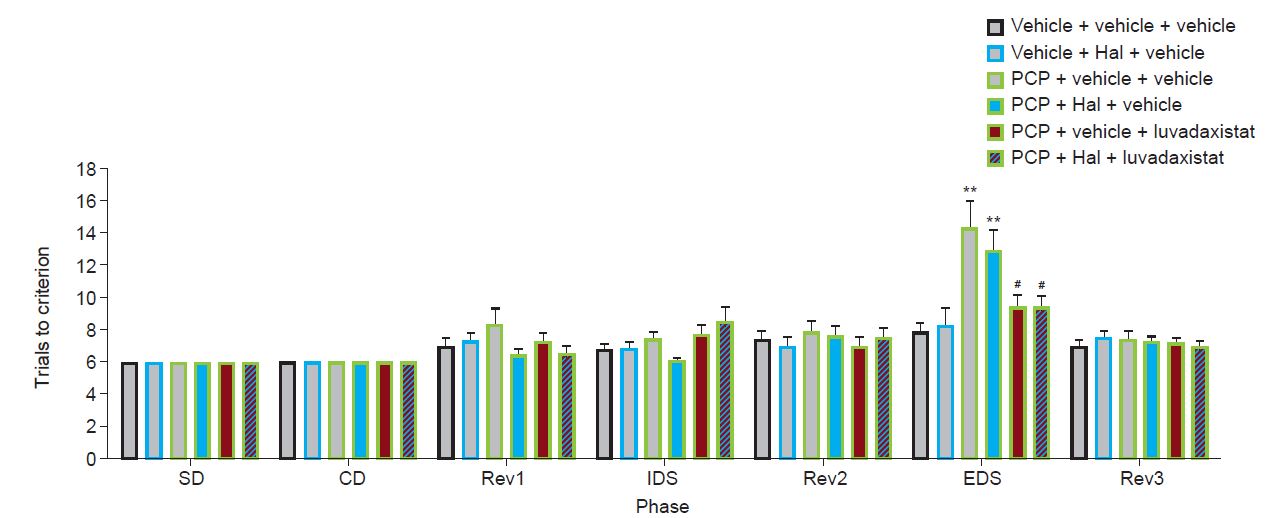


**Supplementary Fig. 4** Time course of the average slope of elicited field responses following LTP induction by a 10×TBS stimulation protocol at hippocampal CA1 synapses from mice 5 h after a single oral administration of VEH, TAK-831 10 mpk, TAK-831 1 mpk, or TAK-831 0.1 mpk. Time point 0 represents delivery of 10 TBS. Slopes of fEPSP are normalized to baseline and plotted against time. fEPSP, field excitatory postsynaptic potential; LTP, long-term potentiation; TBS, theta burst stimulation; VEH, vehicle


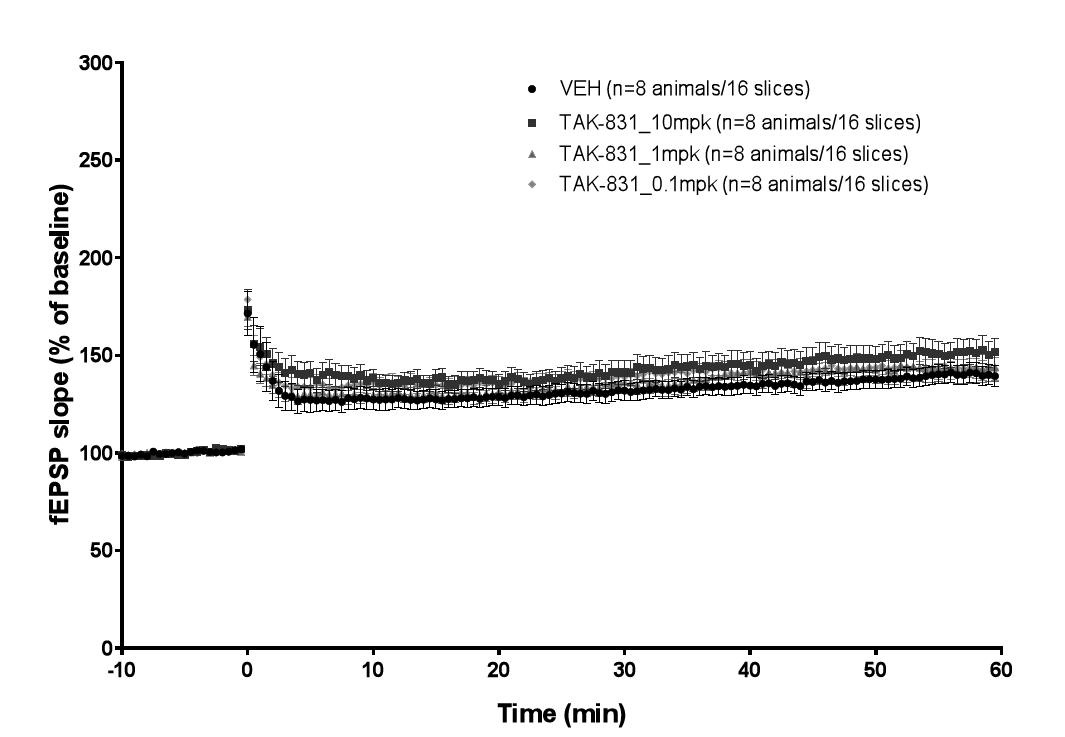


**Supplementary Fig. 5** Examples of recorded fEPSPs during baseline (black) and post LTP induction 40–60 min (gray) following LTP induction by a 10×TBS stimulation protocol at hippocampal CA1 synapses from mice 5 h after a single oral administration of vehicle, TAK-831 10 mpk, TAK-831 1 mpk, or TAK-831 0.1 mpk. fEPSP, field excitatory postsynaptic potential; LTP, long-term potentiation; TBS, theta burst stimulation


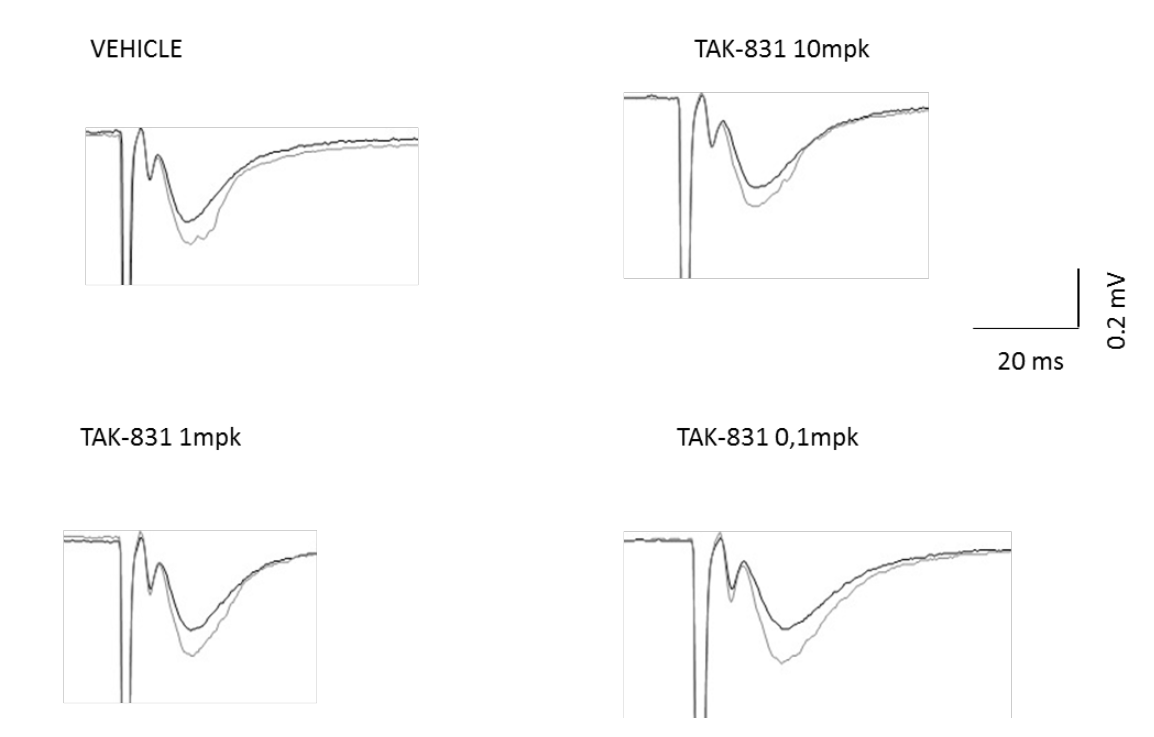


**Supplementary Fig. 6** Time course of the average slope of elicited field responses following LTP induction by a 10×TBS stimulation protocol at hippocampal CA1 synapses from mice 5 h after 14-days oral administration of VEH, TAK-831 10 mg/kg, TAK-831 1 mg/kg, TAK-831 0.1 mg/kg, or TAK-831 0.01 mg/kg. Time point 0 represents delivery of 10×TBS. Slopes of fEPSP are normalized to baseline and plotted against time. fEPSP, field excitatory postsynaptic potential; LTP, long-term potentiation; TBS, theta burst stimulation; VEH, vehicle


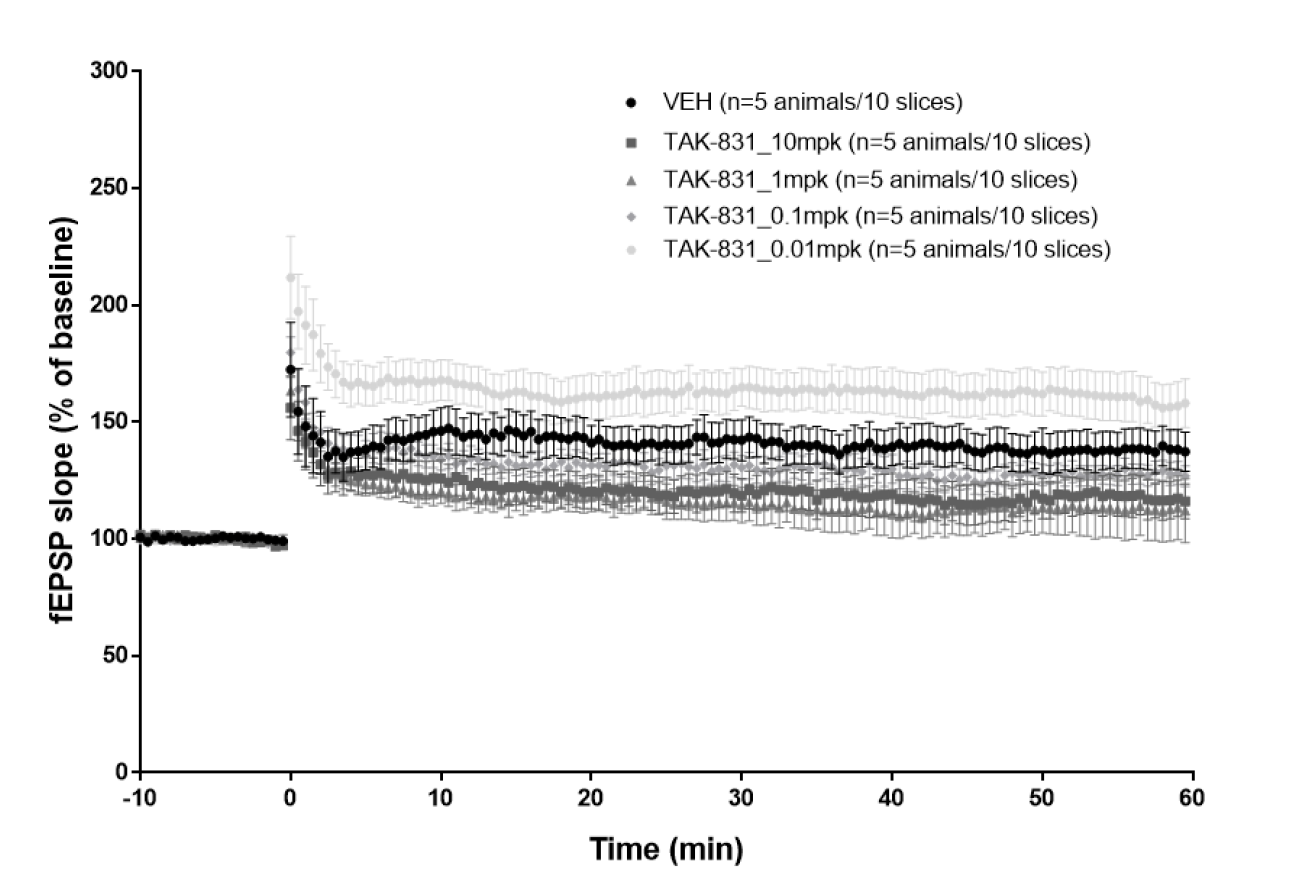


**Supplementary Fig. 7** Examples of recorded fEPSPs during baseline (black) and post LTP induction 40–60 min (gray) following LTP induction by a 10×TBS stimulation protocol at hippocampal CA1 synapses from mice 5 h after a single oral administration of vehicle, TAK-831 10 mg/kg, TAK-831 1 mg/kg, TAK-831 0.1 mg/kg, or TAK-831 0.01 mg/kg. fEPSP, field excitatory postsynaptic potential; LTP, long-term potentiation; TBS, theta burst stimulation


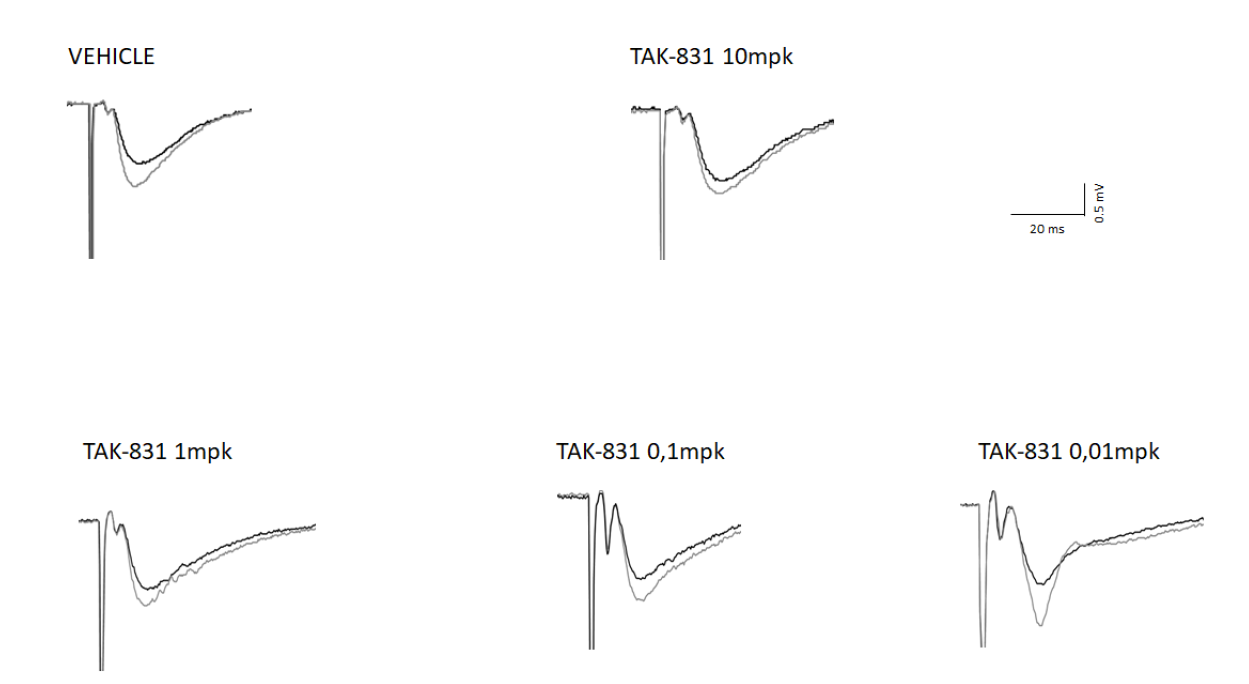

Supplement: Supplementary file 1 — Supplementary file1 (DOCX 776 KB) [file 11064_2023_3956_MOESM1_ESM.docx]
